# Supplementary material for: Synthesis of Natural (−)-Antrocin and Its Enantiomer via Stereoselective Aldol Reaction
Source: Molecules. 2020 Feb 14;25(4):831. doi: 10.3390/molecules25040831 (PMC7070359; doi:10.3390/molecules25040831)
Supplement: Supplementary file 1 [file molecules-25-00831-s001.pdf]

Supporting information

Synthesis of natural (–)-antrocin and its enantiomer *via* stereoselective aldol reaction

Venkatachalam Angamuthu[1] and Dar-Fu Tai[2]*

[1]Department of Chemistry, Pondicherry University, Puducherry, India; E-mail: venkatachalam_83@yahoo.co.in

[2] Department of chemistry, National Dong Hwa University, Hualien, Taiwan (R.O.C); E-Mail: dftai@gms.ndhu.edu.tw

*Correspondence: E-mail: dftai@gms.ndhu.edu.tw

Table of the content

Figure S1 ^{1}H NMR for the compound **4.** **S4**

Figure S2 ^{13}C NMR for the compound **4**.. **S5**

Figure S3 HRMS (ESI) for the compound **4**.. **S6**

Figure S4 ^{1}H NMR for the compound **5** **S7**

Figure S5 ^{13}C NMR for the compound **5** **S8**

Figure S6 HRMS (ESI) for the compound **5**.. **S9**

Figure S7 ^{1}H NMR for the compound **6** **S10**

Figure S8 ^{13}C NMR for the compound **6** **S11**

Figure S9 HRMS (ESI) for the compound **6**.. **S12**

Figure S10 ^{1}H NMR for the compound **trans(±)-7** **S13**

Figure S11 ^{13}C NMR for the compound **trans(±)-7** **S14**

Figure S12 HRMS (ESI) for the compound **trans(±)-7**..**S15**

Figure S13 ^{1}H NMR for the compound cis**(±)-7**.. **S16**

Figure S14 ^{13}C NMR for the compound cis**(±)-7**..**S17**

Figure S15 ^{1}H NMR for the compound **(-)-8**.. **S18**

Figure S16 ^{13}C NMR for the compound **(-)-8**.. **S19**

Figure S17 HRMS (ESI) for the compound **(-)-8** **S20**

Figure S18 ^{1}H NMR for the compound **(+)-9**.. **S21**

Figure S19 ^{13}C NMR for the compound **(+)-9**.. **S22**

Figure S20 HRMS (ESI) for the compound **(+)-9** **S23**

Figure S21 ^{1}H NMR for the compound **(-)-10**.. **S24**

Figure S22 ^{13}C NMR for the compound **(-)-10**.. **S25**

Figure S23 HRMS (ESI) for the compound **(-)-10** **S26**

Figure S24 ^{1}H NMR for the compound (+)-**12**.. **S27**

Figure S25 ^{13}C NMR for the compound (+)-**12**.. **S28**

Figure S26 HRMS (ESI) for the compound (+)-**12** **S29**

Figure S27 ^{1}H NMR for the compound (+)-**13**.. **S30**

Figure S28 ^{13}C NMR for the compound (+)-**13**.. **S31**

Figure S29 2D NOESY for the compound **(+)-13**.. **S32**

Figure S30 HRMS (ESI) for the compound (+)-**13** **S33**

Figure S31 ^{1}H NMR for the compound **(-)-14**.. **S34**

Figure S32 ^{13}C NMR for the compound **(-)-14**.. **S35**

Figure S33 HRMS (ESI) for the compound **(-)-14** **S36**

Figure S34 ^{1}H NMR for the compound (-)-**1a**.. **S37**

Figure S35 ^{13}C NMR for the compound (-)-**1a**.. **S38**

Figure S36 DEPT 135, 90 and 45 combined spectra for the compound (-)-**1** **S39**

Figure S37 HRMS (ESI) for the compound **(-)-1** S40

Figure S38 ^{1}H NMR for the compound **15** and **16** mixture S41

Figure S39 ^{1}H NMR for the compound **15** S42

Figure S40 ^{13}C NMR for the compound **15** S43

Figure S41 ^{1}H NMR for the compound **16** S44

Crystallographic data for trans cyanobicyclic ketone, (–)-7… S45

Crystallographic data for ketol diastreomer, (–)-8… S50

Crystallographic data for lactone (–)-14… S62

Figure S1

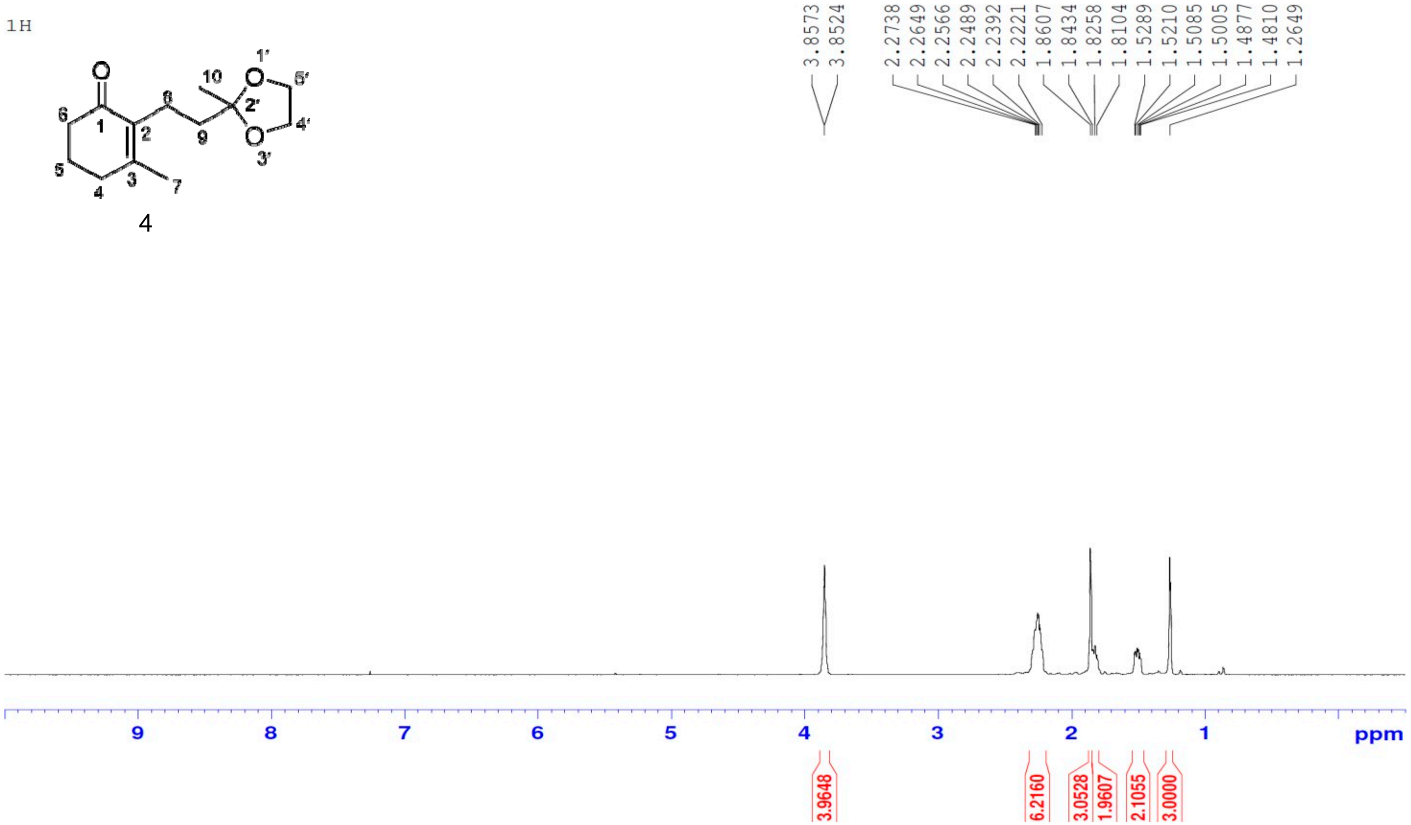

^{1}H NMR of compound **4** (400 MHz, $CDCl_3$)

Figure S2

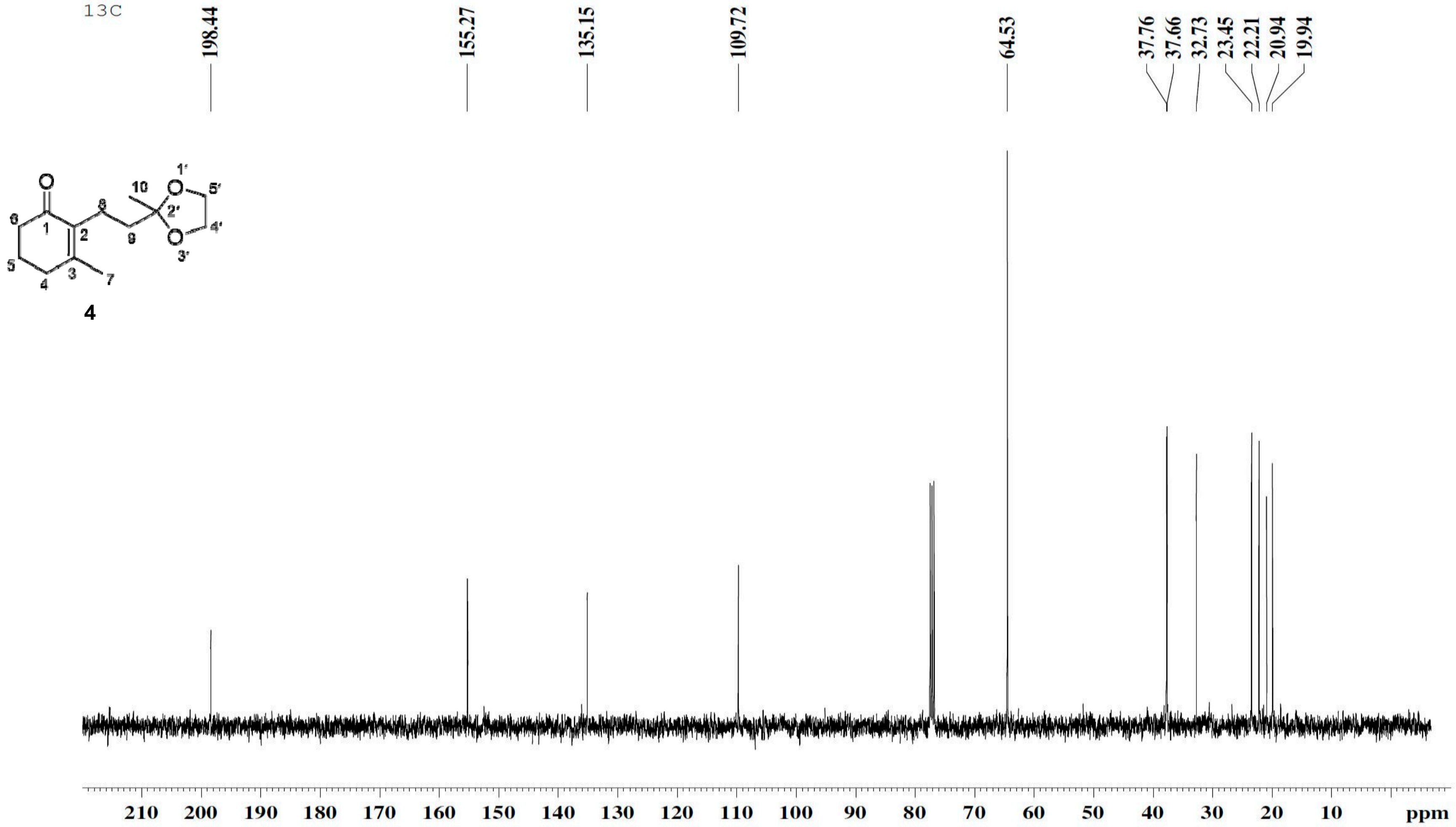

^{13}C NMR of compound **4** (100 MHz, $CDCl_3$)

Figure S3

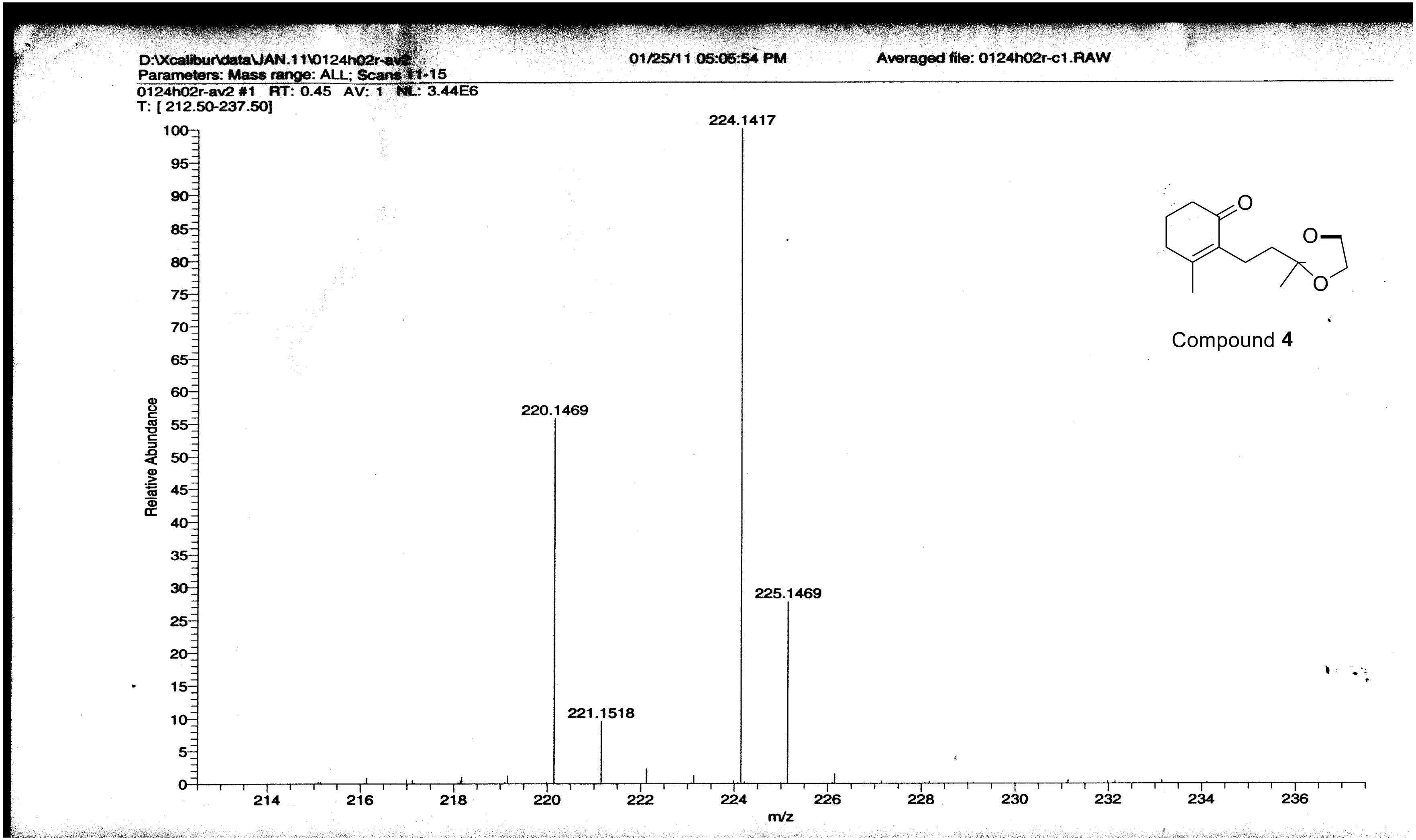

HRMS (ESI) of compound **4**

Figure S4

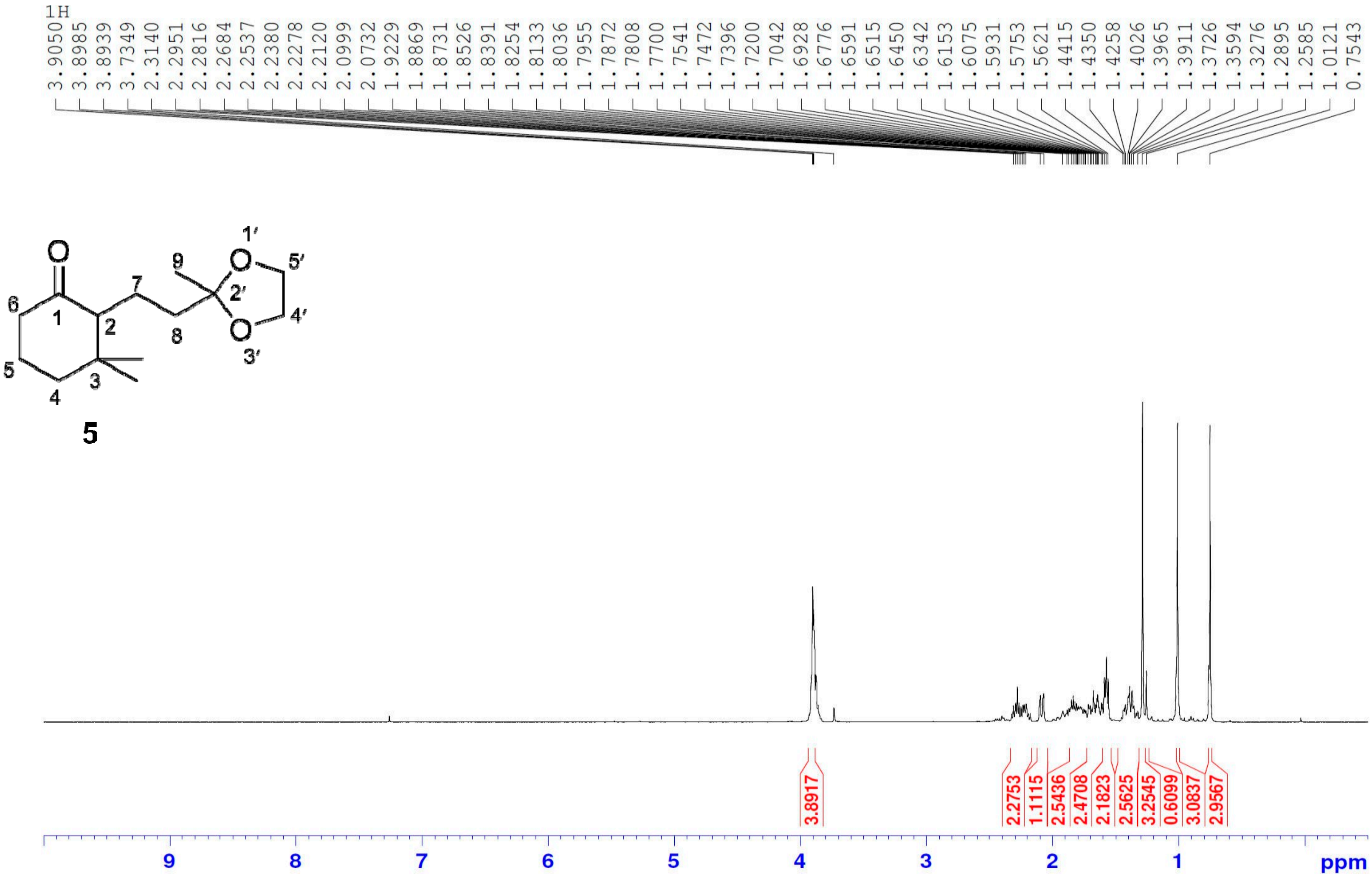

5

^{1}H NMR of compound **5** (400 MHz, $CDCl_3$)

Figure S5

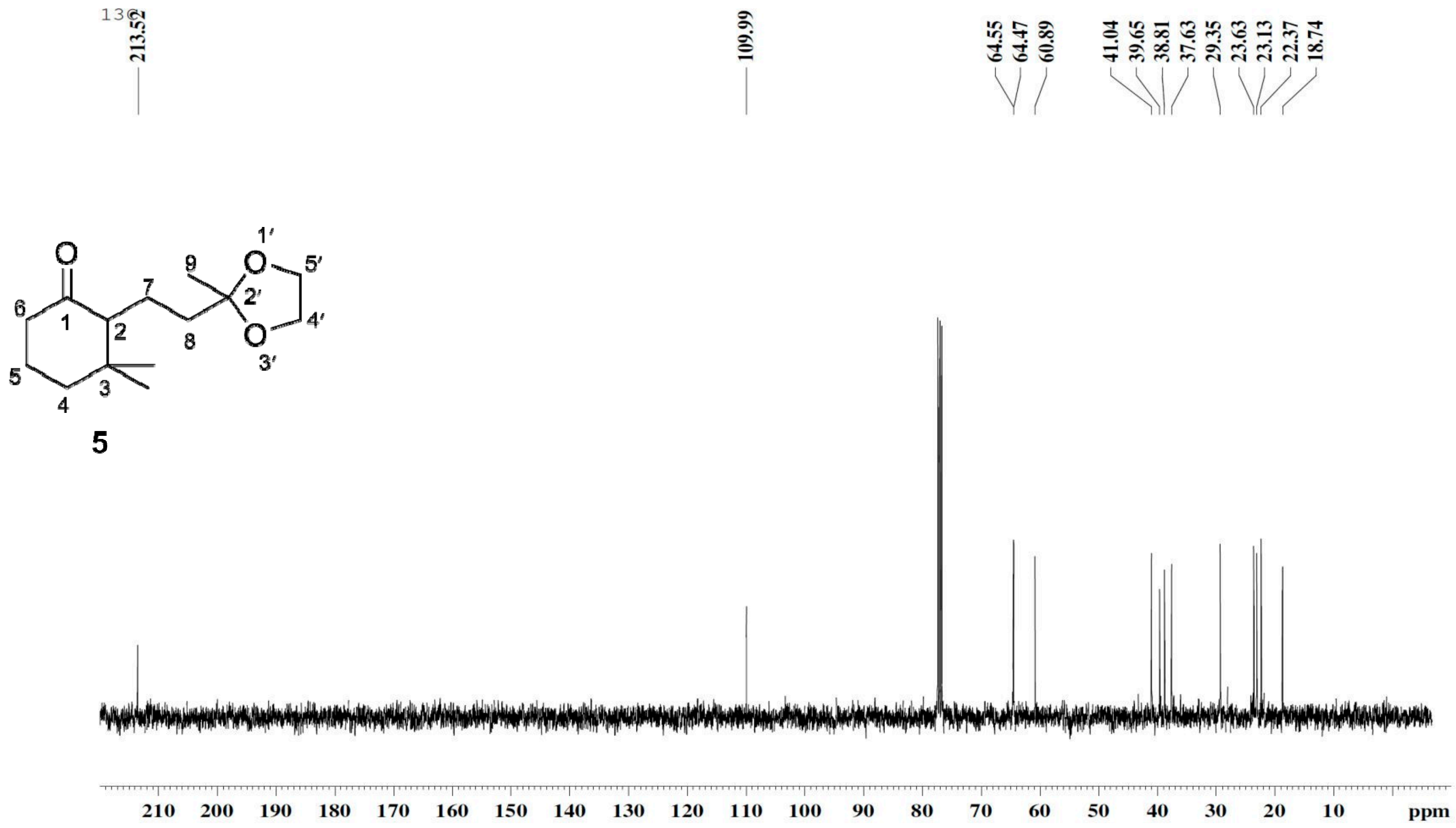

5

^{13}C NMR of compound **5** (100 MHz, $CDCl_3$)

Figure S5

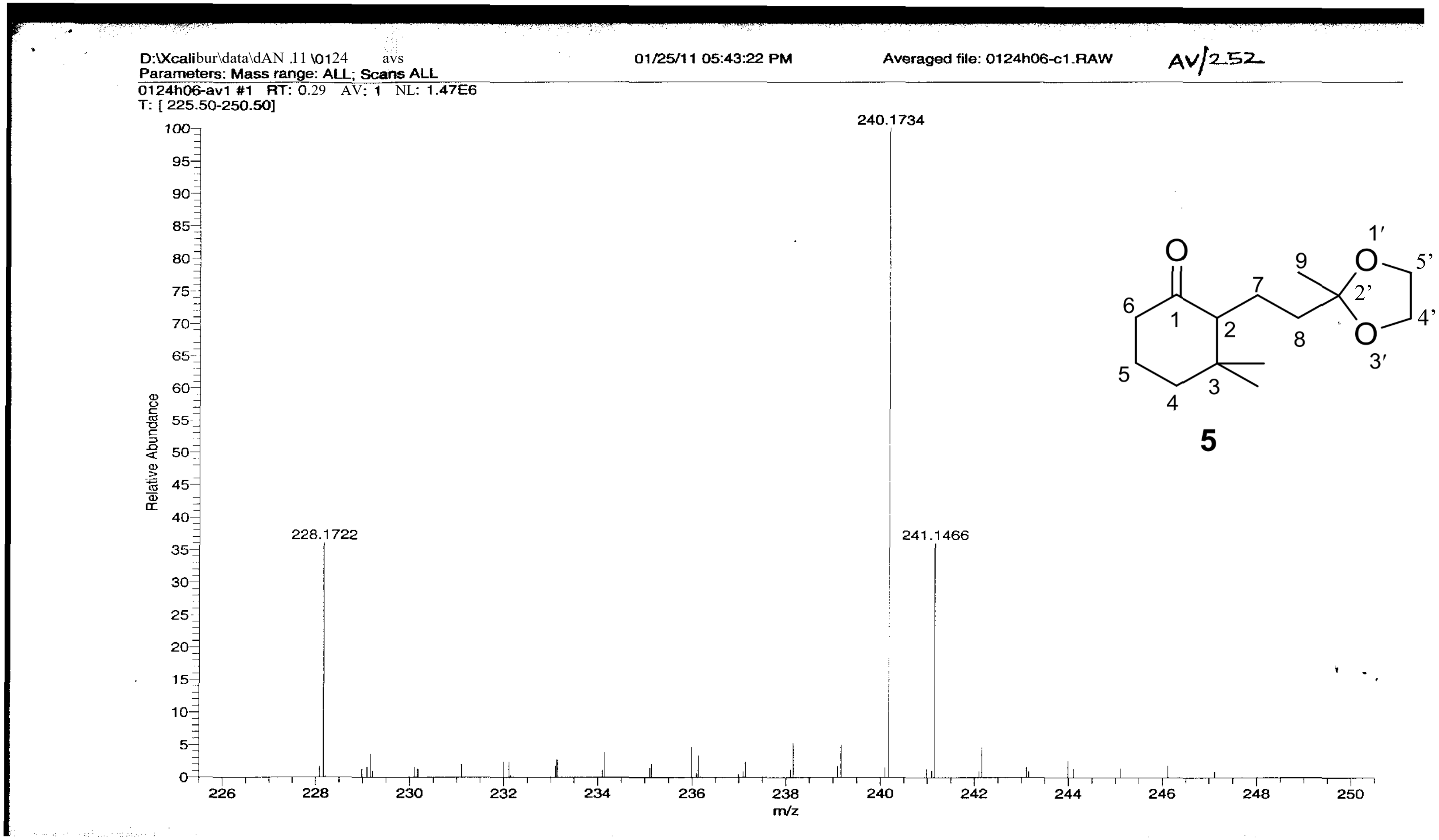

HRMS (ESI) of compound 5

Figure S7

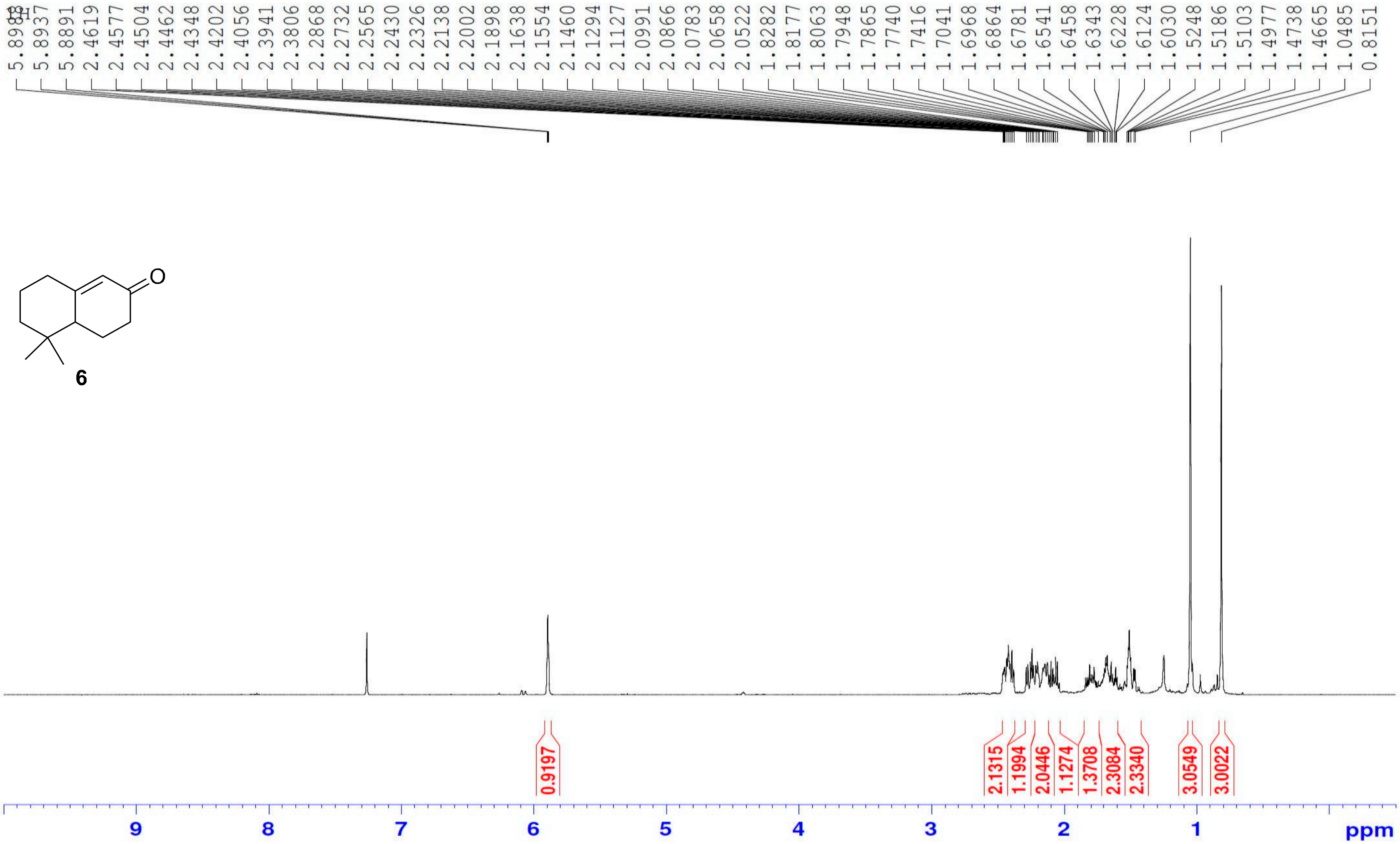

^{1}H NMR of compound **6** (400 MHz, $CDCl_3$)

Figure S8

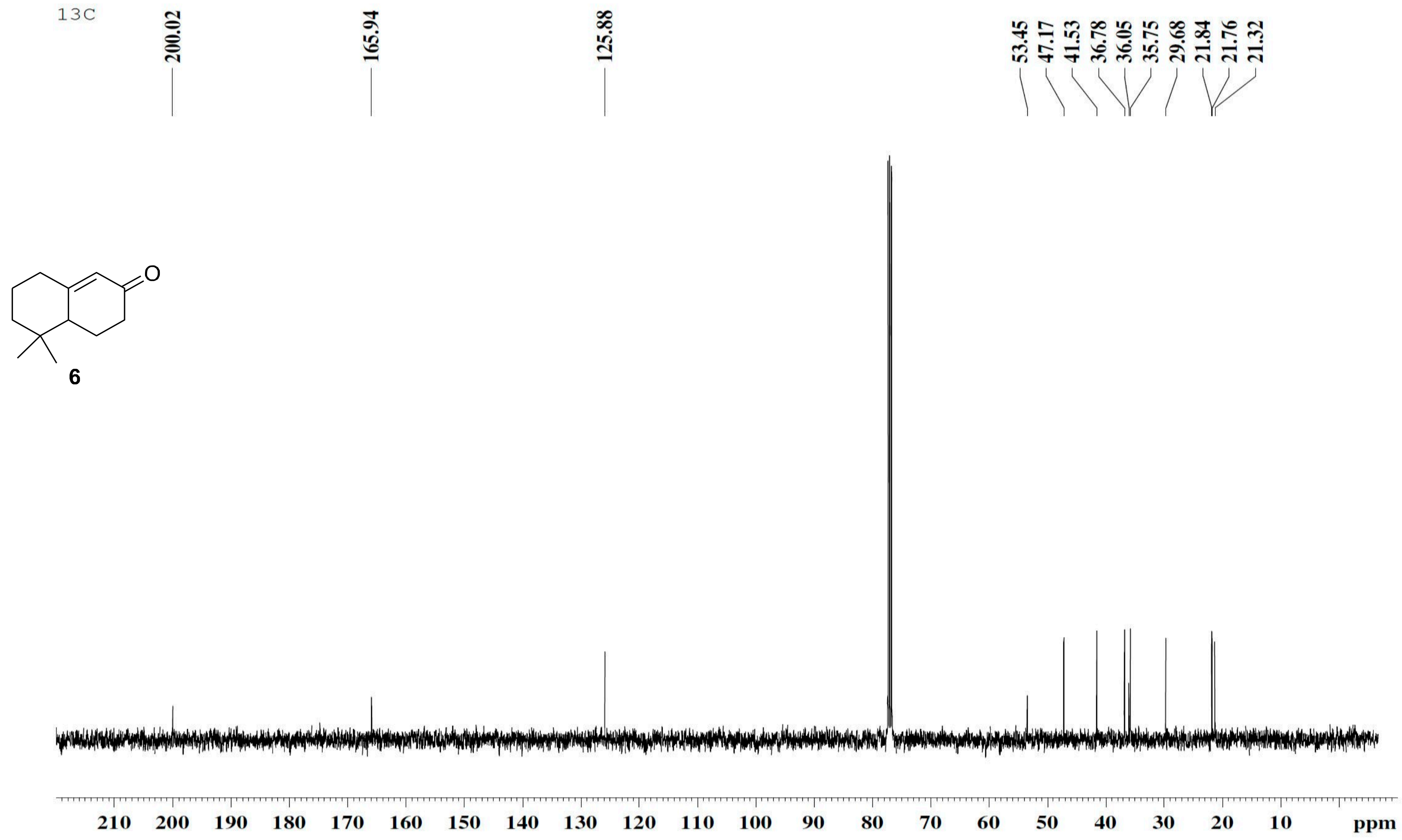

^{13}C NMR of compound **6** (100 MHz, $CDCl_3$)

Figure S9

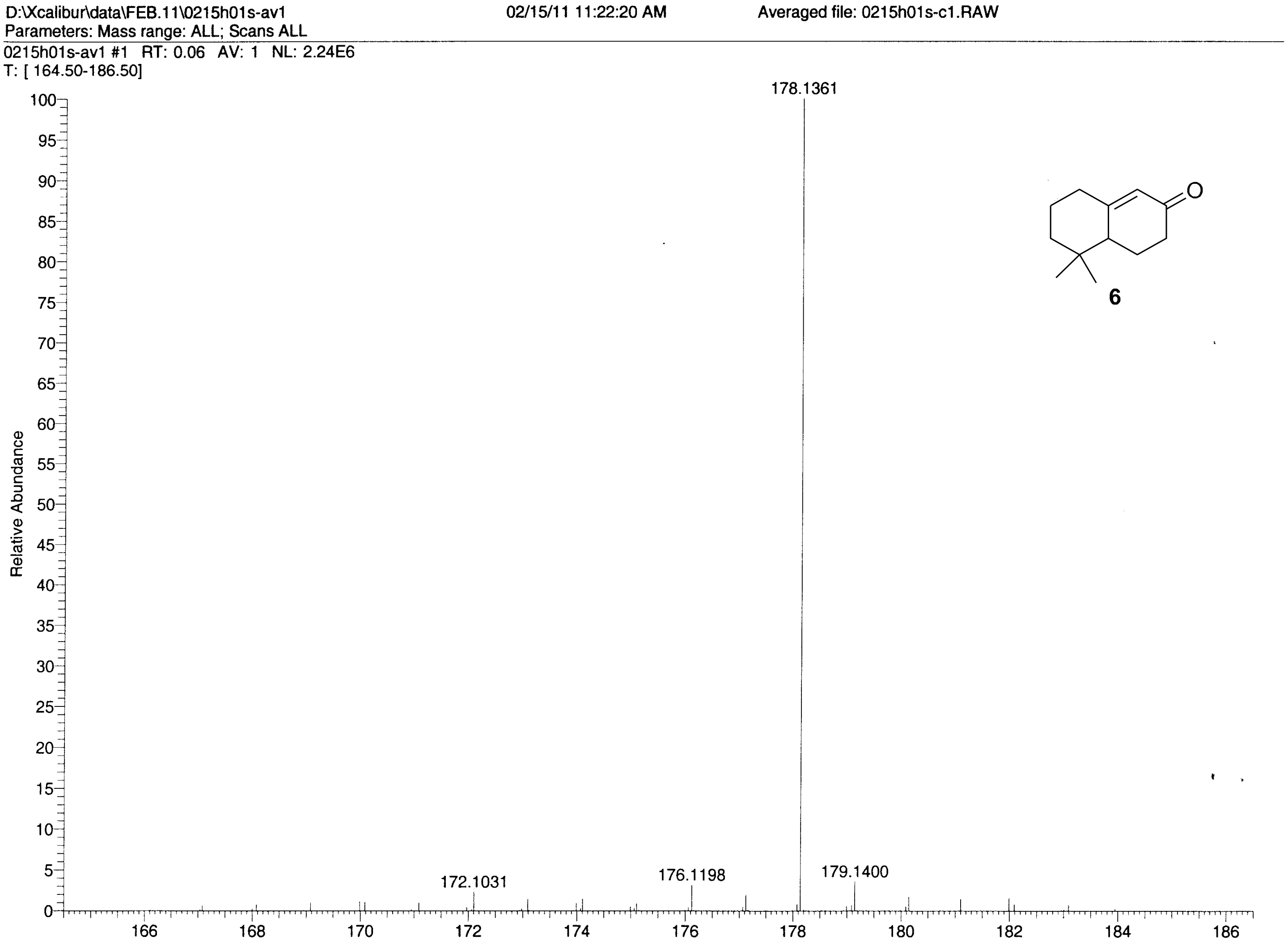

HRMS (ESI) of compound **6**

Figure S10

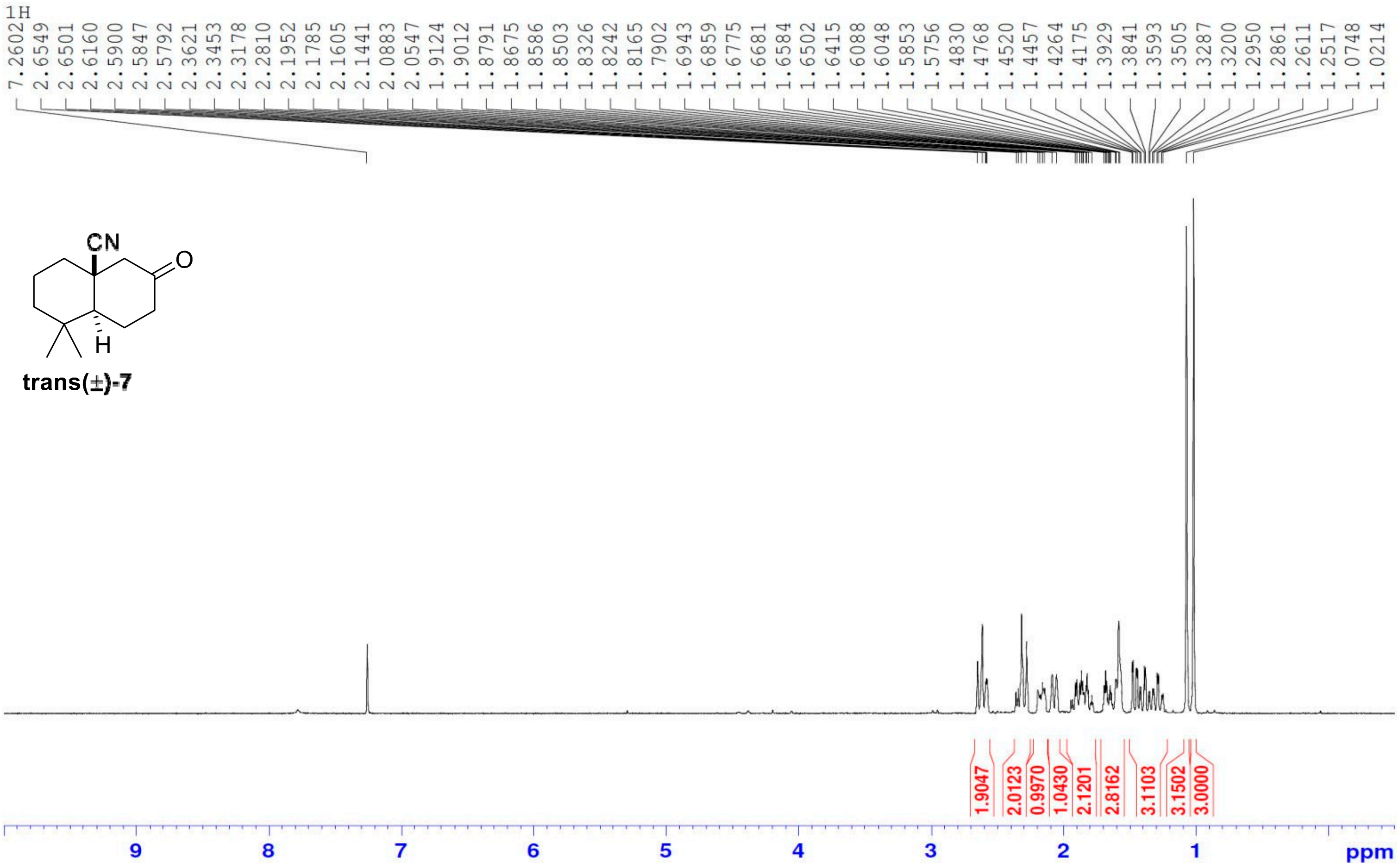

^{1}H NMR of compound trans-**(±)-7** (400 MHz, $CDCl_3$)

Figure S11

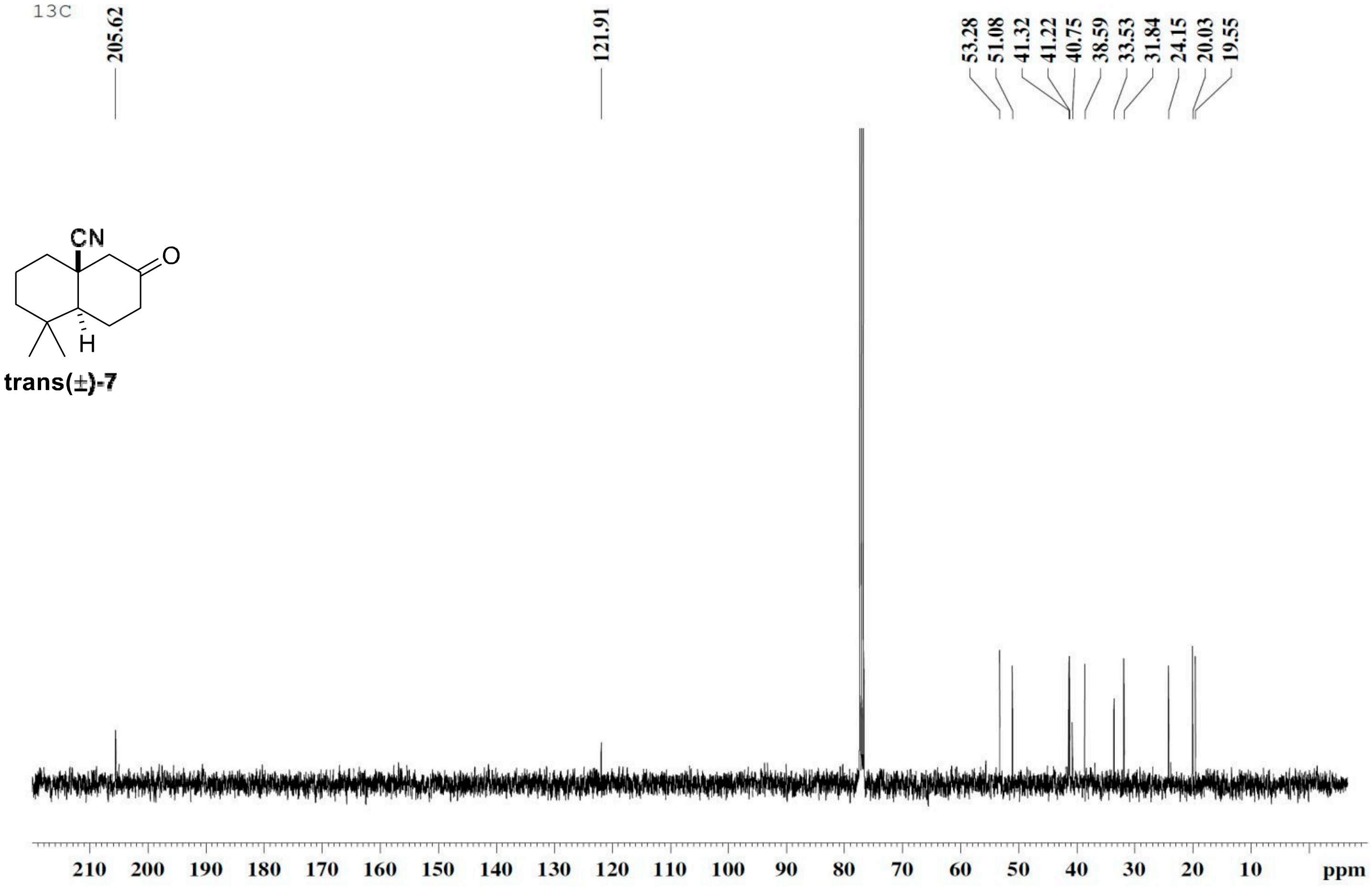

trans(±)-7

^{13}C NMR of compound trans-**(±)-7** (100 MHz, $CDCl_3$)

Figure S12

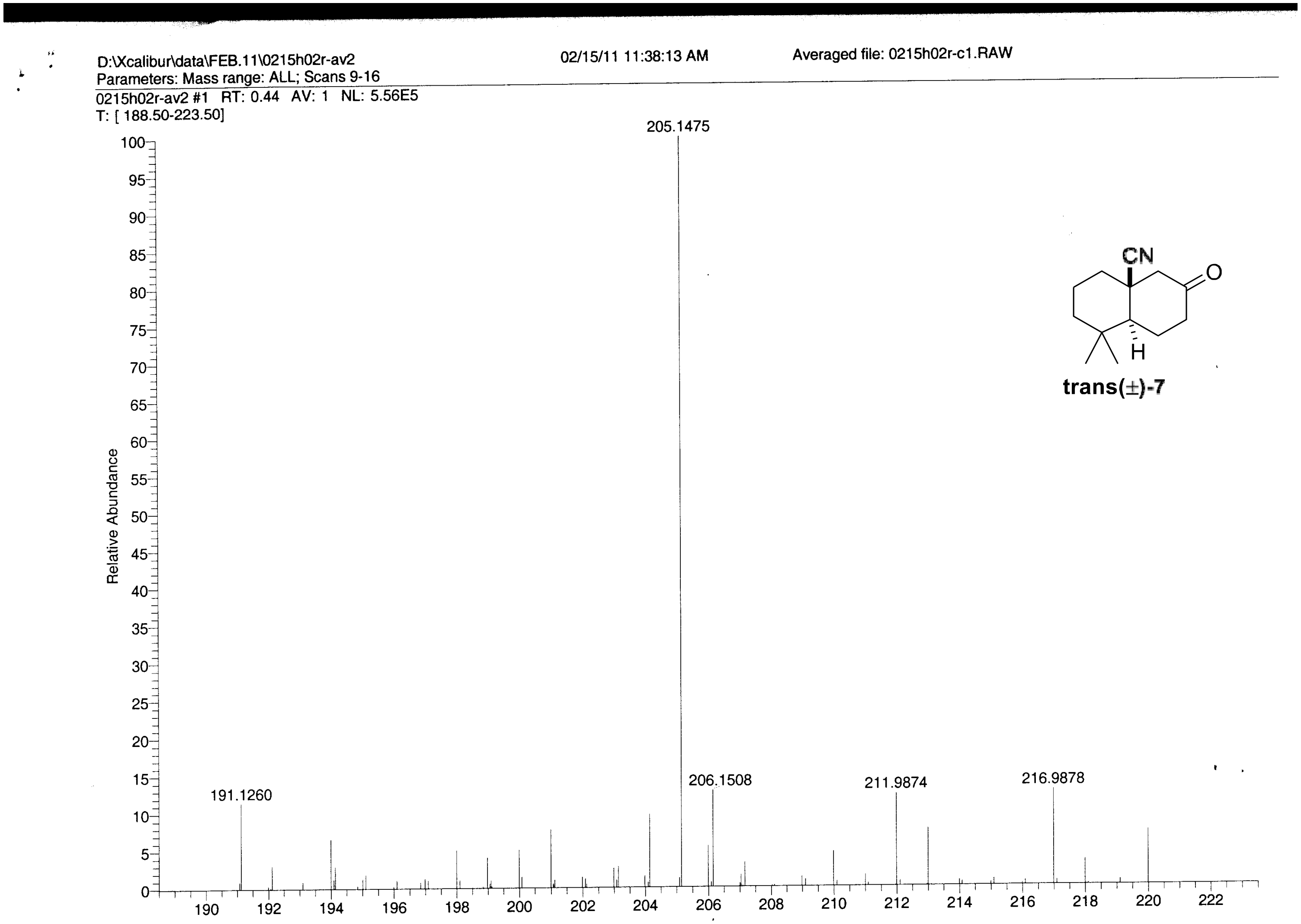

HRMS (ESI) of compound trans-**(±)-7**

Figure S13

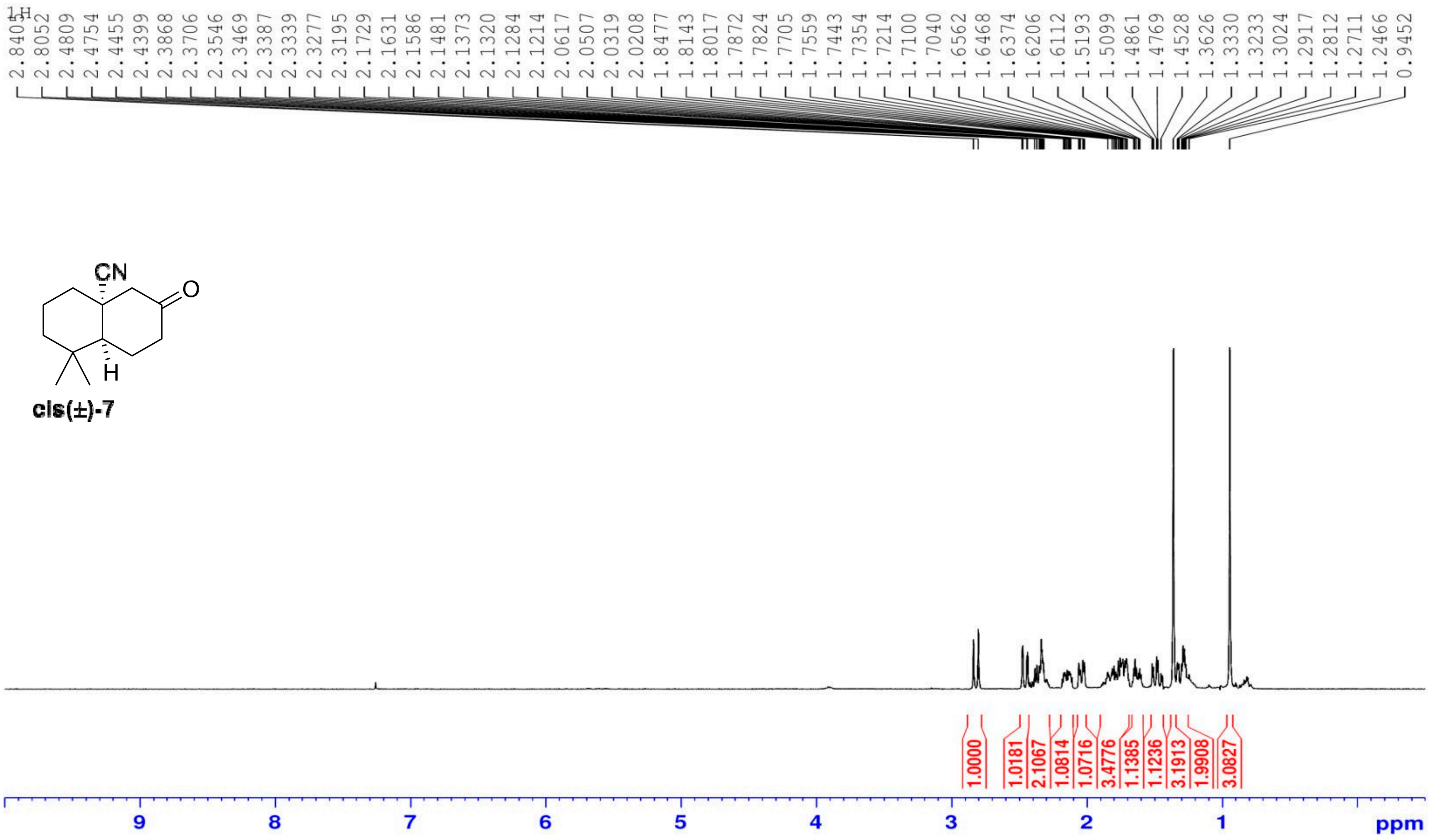

^{1}H NMR of compound cis-**(±)-7** (400 MHz, $CDCl_3$)

Figure S14

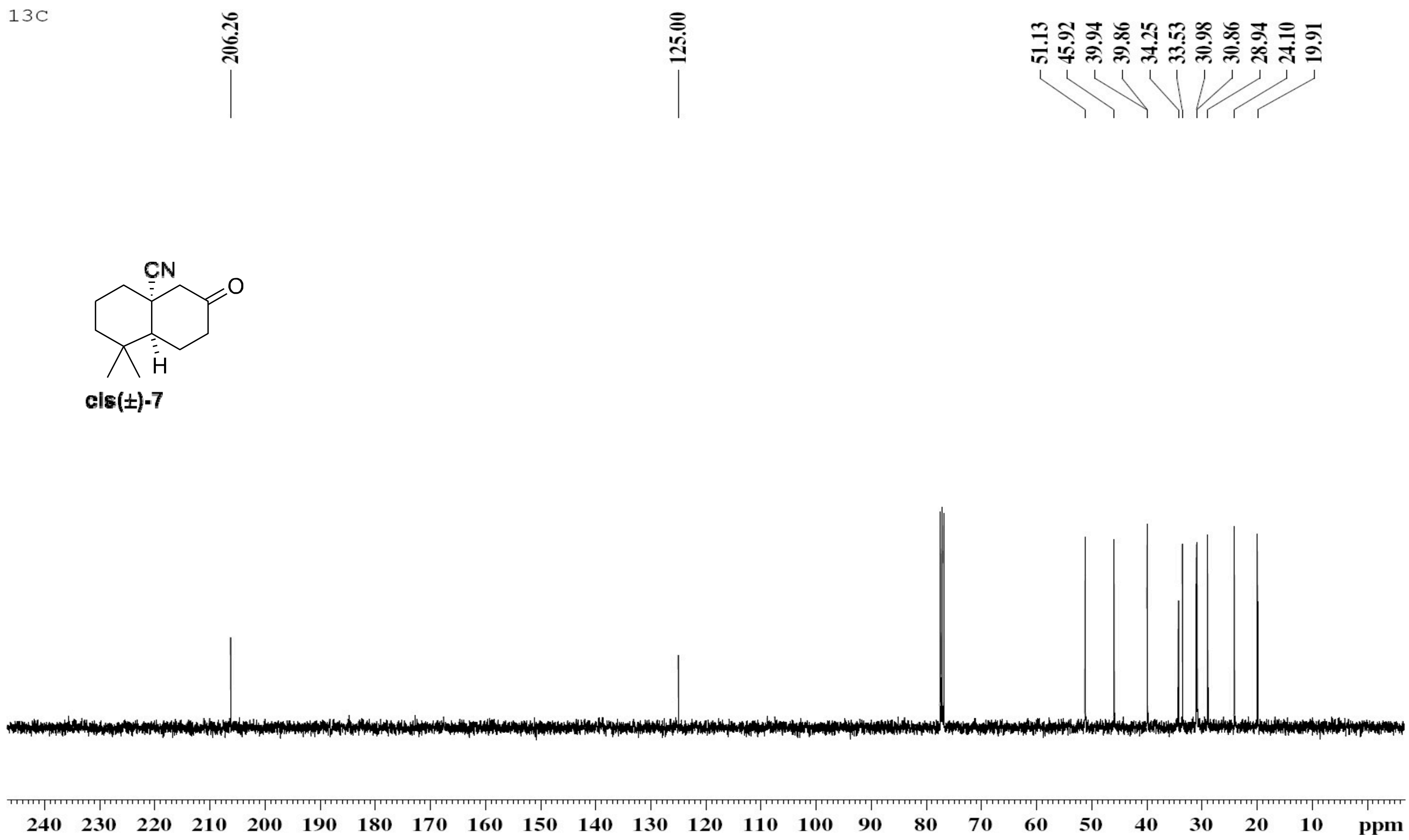

cis(±)-7

^{13}C NMR of compound cis-**(±)-7** (100 MHz, $CDCl_3$)

Figure S15

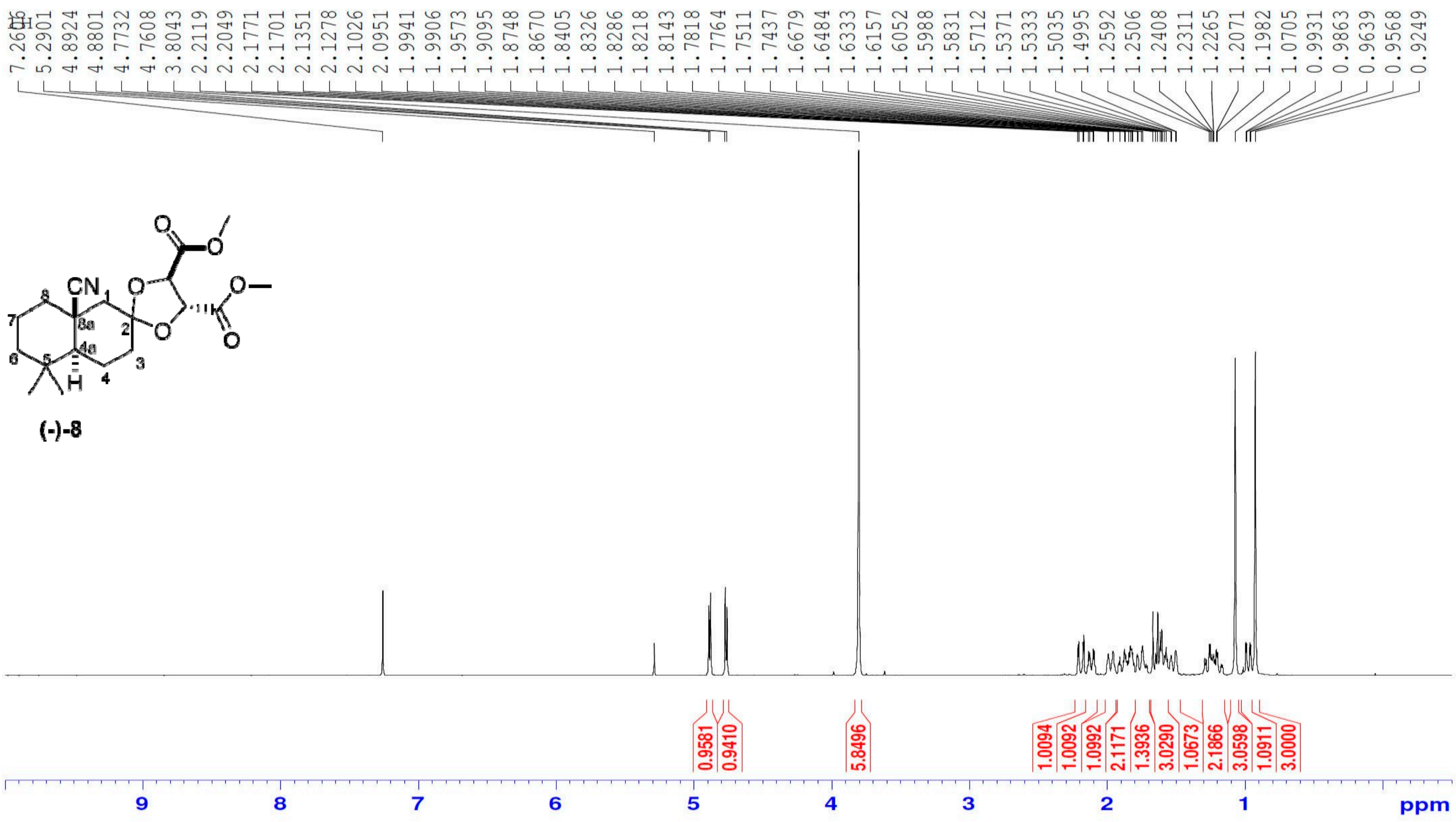

^1H NMR of compound **(-)-8** (400 MHz, $CDCl_3$)

Figure S16

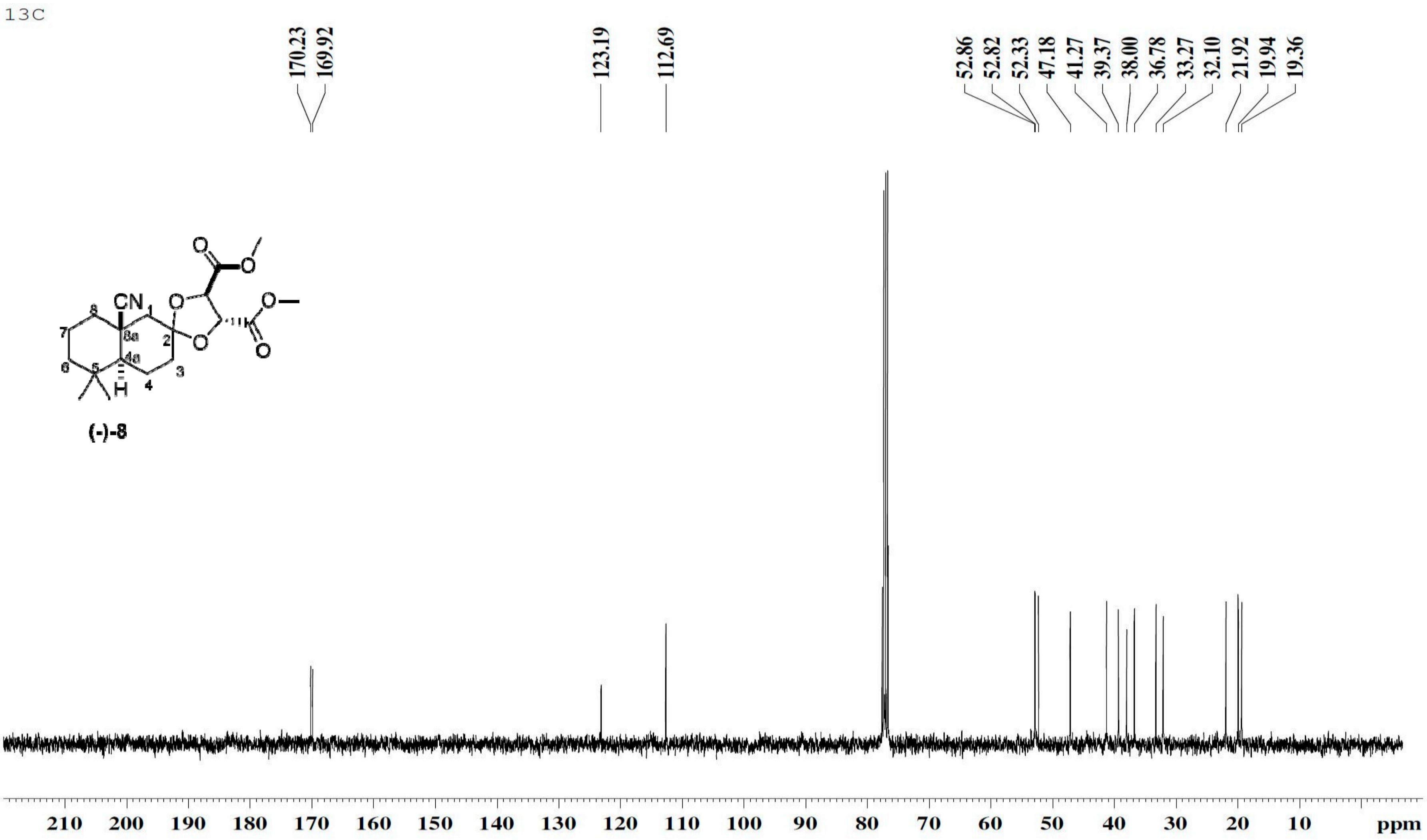

^{13}C NMR of compound **(-)-8** (100 MHz, $CDCl_3$)

Figure S17

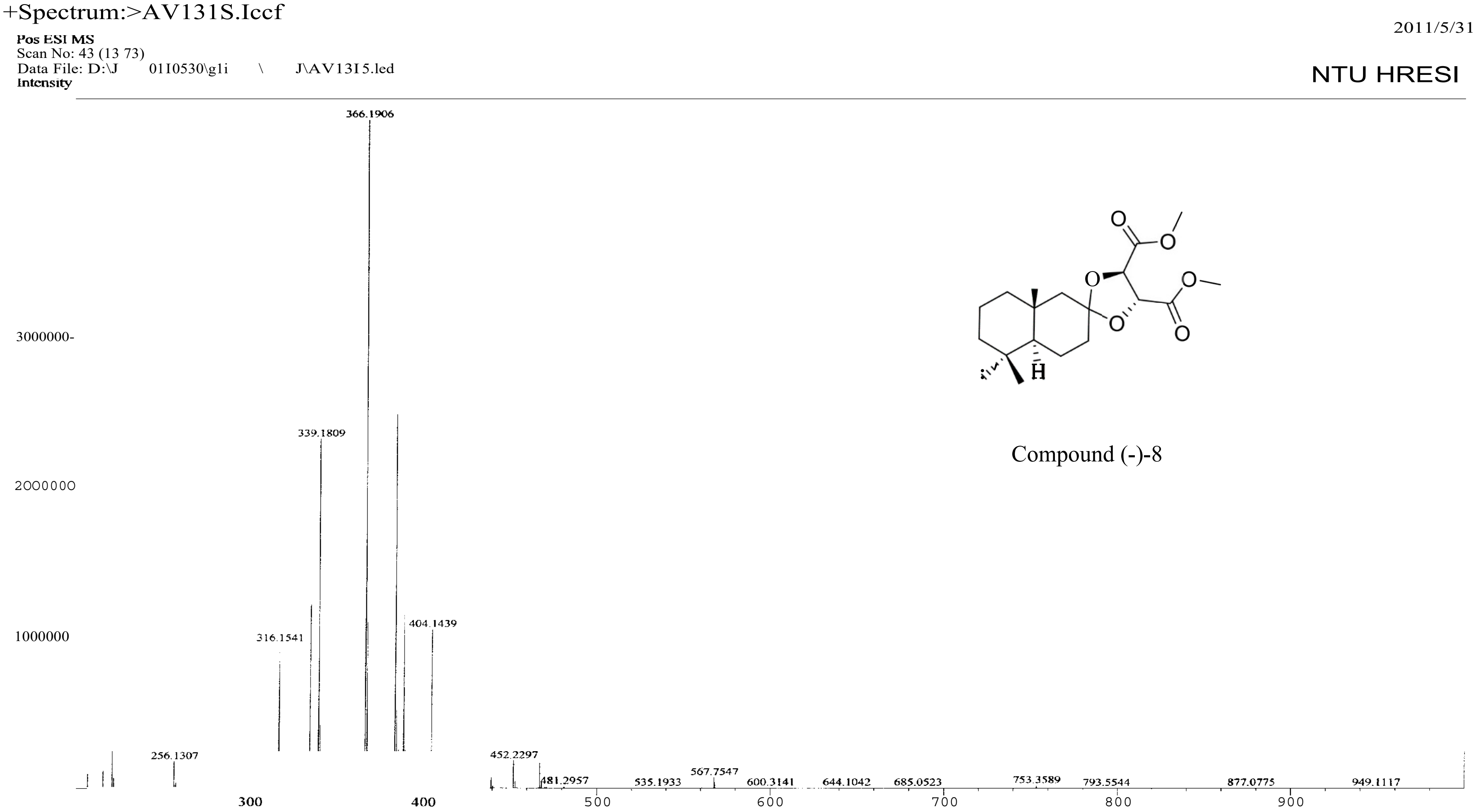

Compound (-)-8

HRMS (ESI) of compound (-)-8

Figure S18

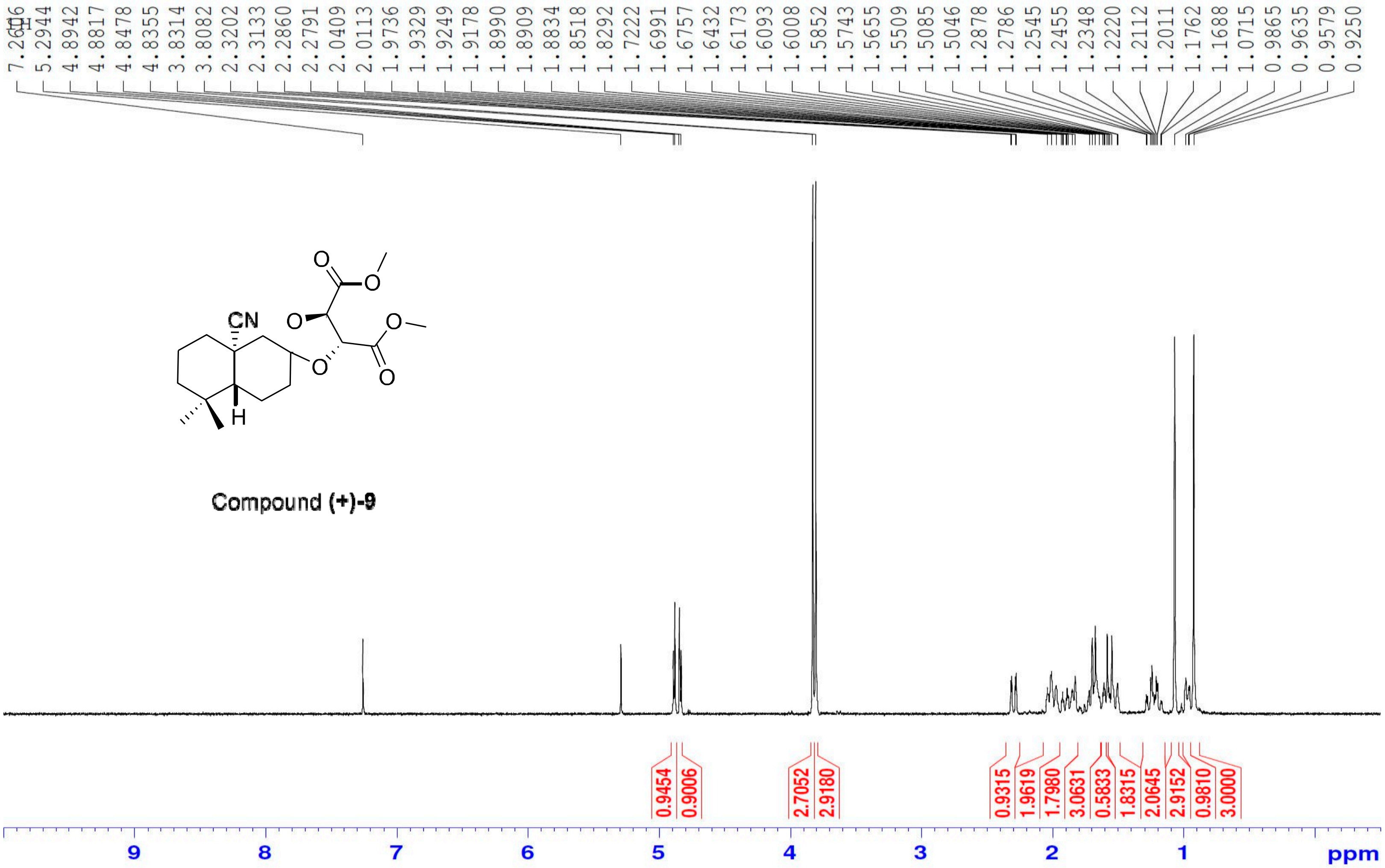

^{1}H NMR of compound (+)**-9** (400 MHz, $CDCl_3$)

Figure S19

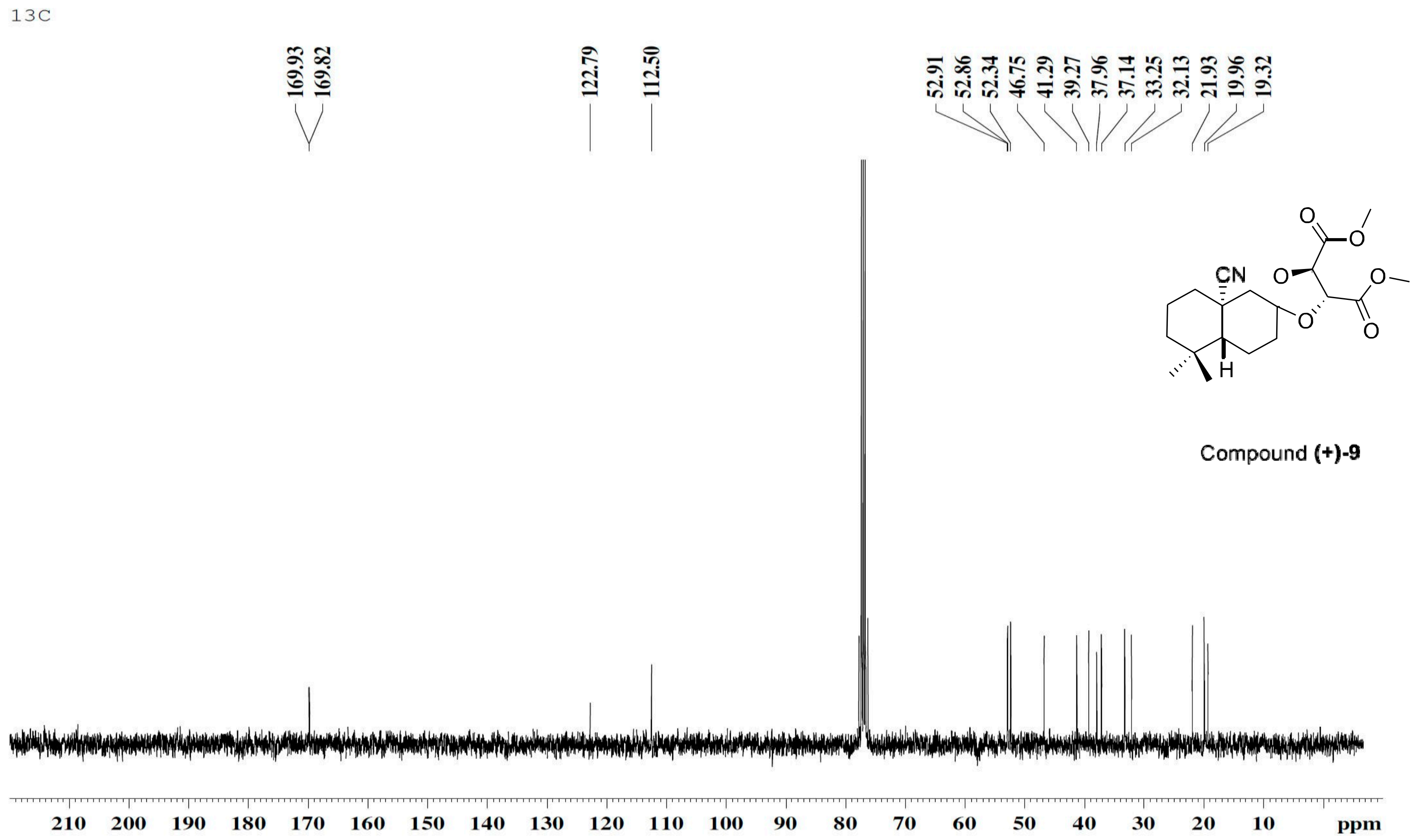

^{13}C NMR of compound (+)-**9** (100 MHz, $CDCl_3$)

Figure 520

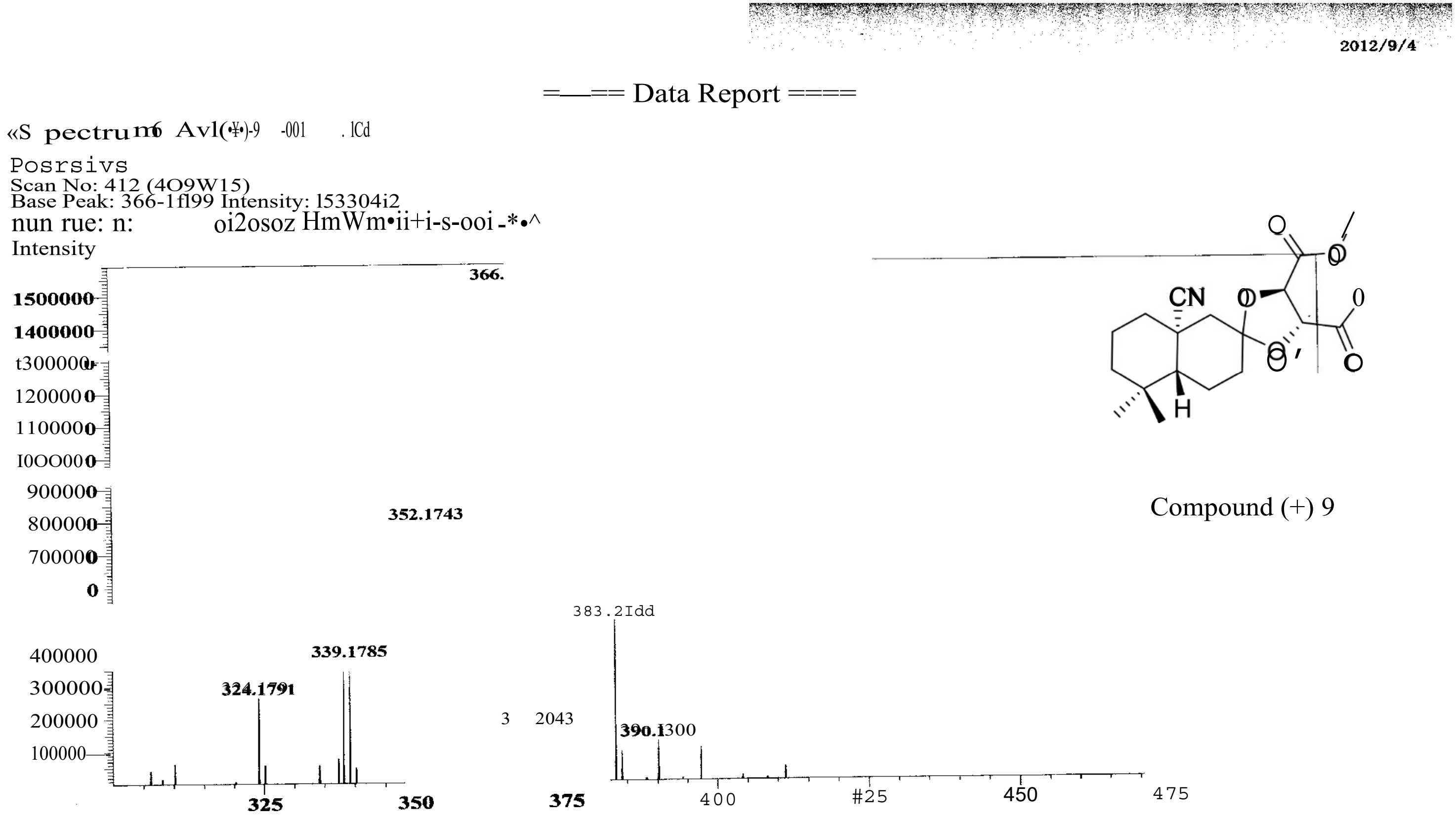

HRMS (ESI) of compound (+)-9

Figure S21

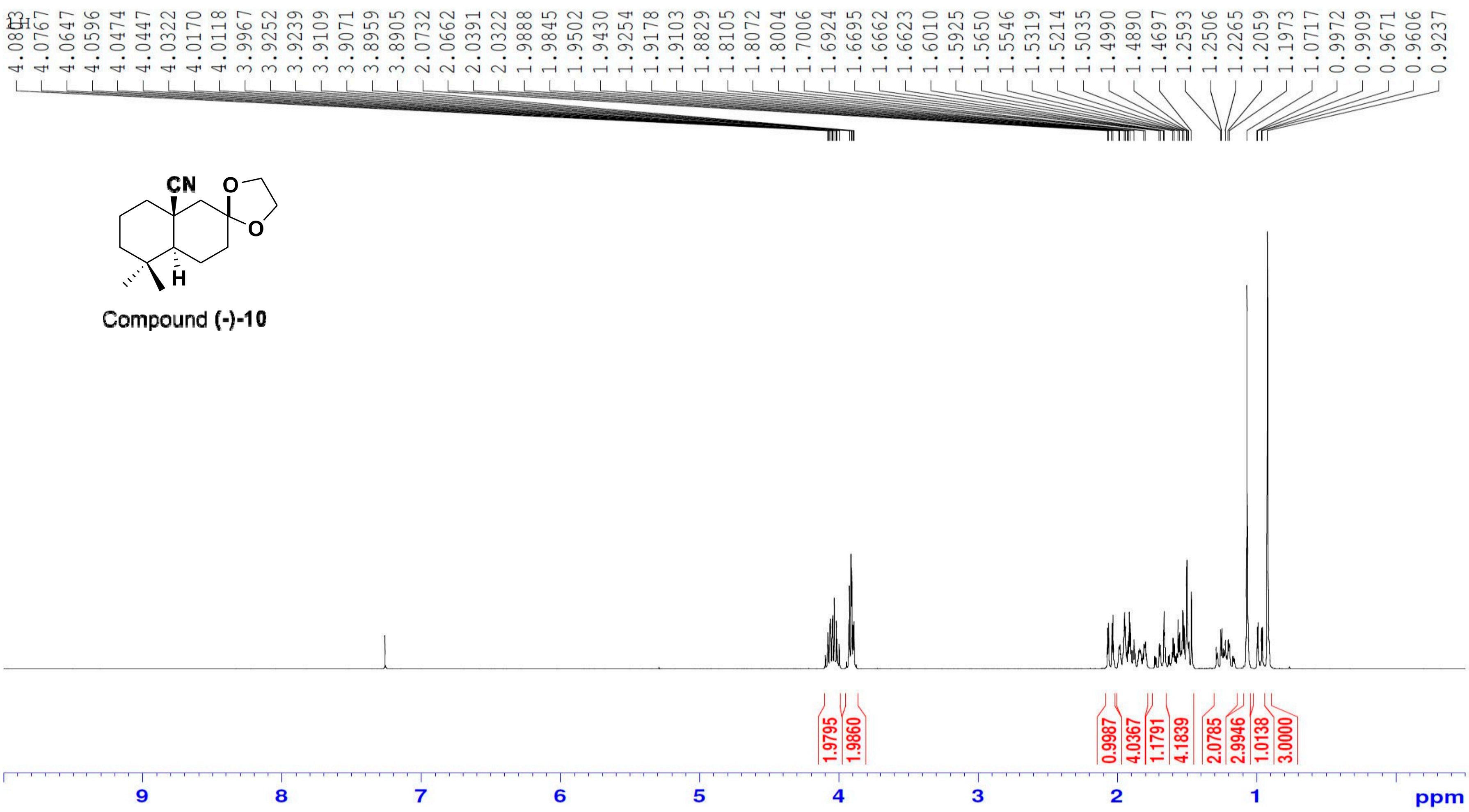

^{1}H NMR of compound (-)-**10** (400 MHz, $CDCl_3$)

Figure S22

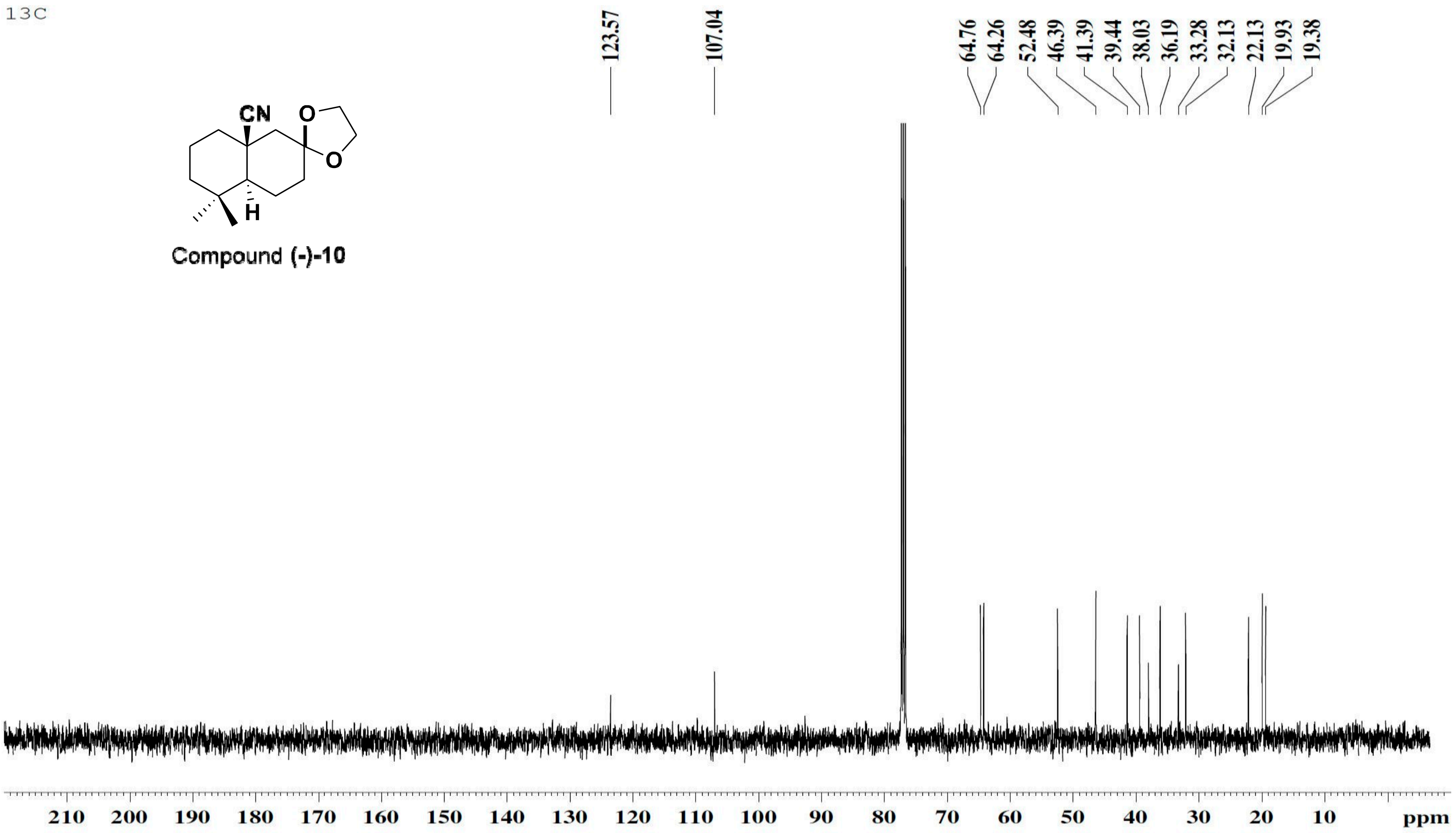

^{13}C NMR of compound **(-)-10** (100 MHz, $CDCl_3$)

Figure S23

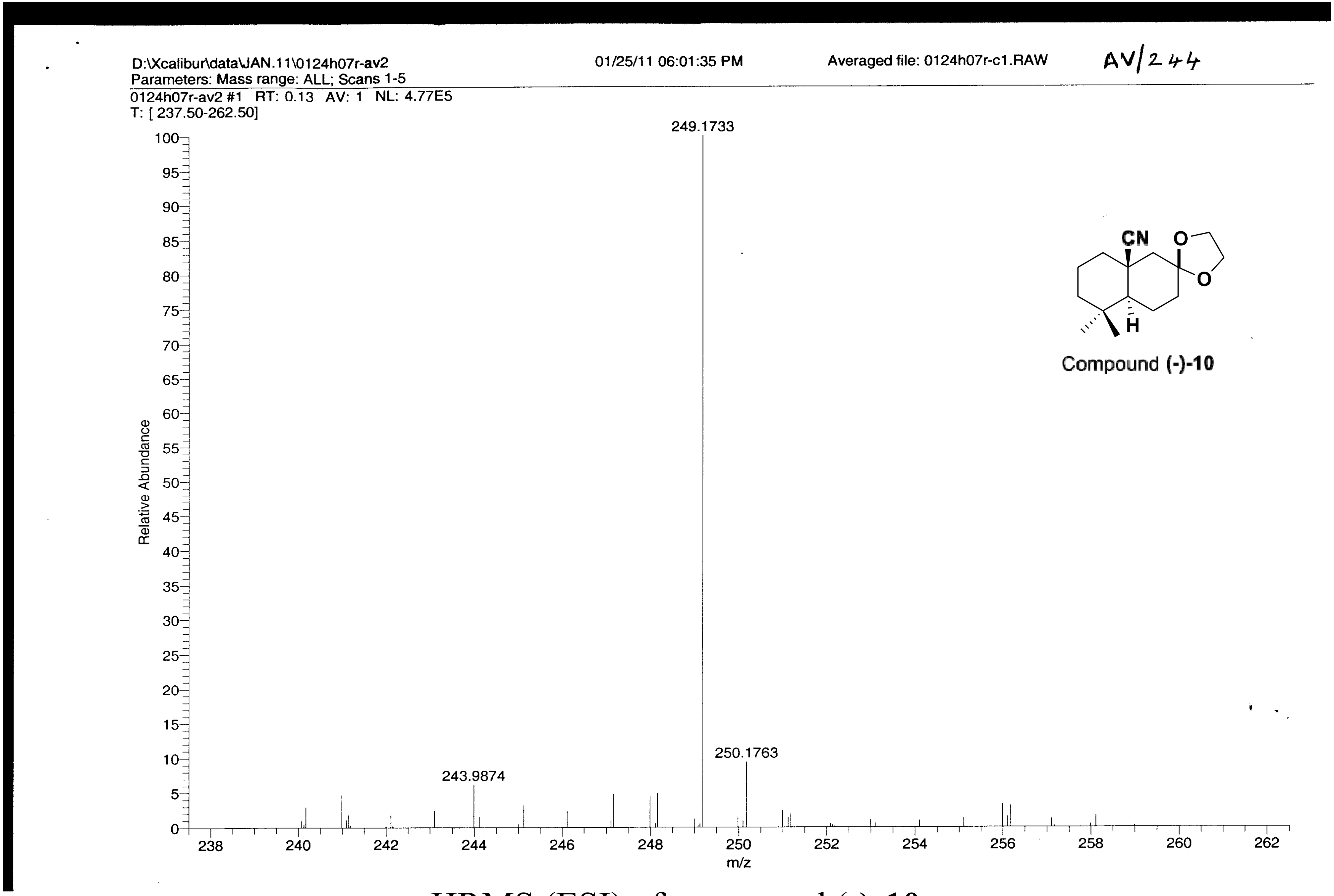

HRMS (ESI) of compound **(-)-10**

Figure S24

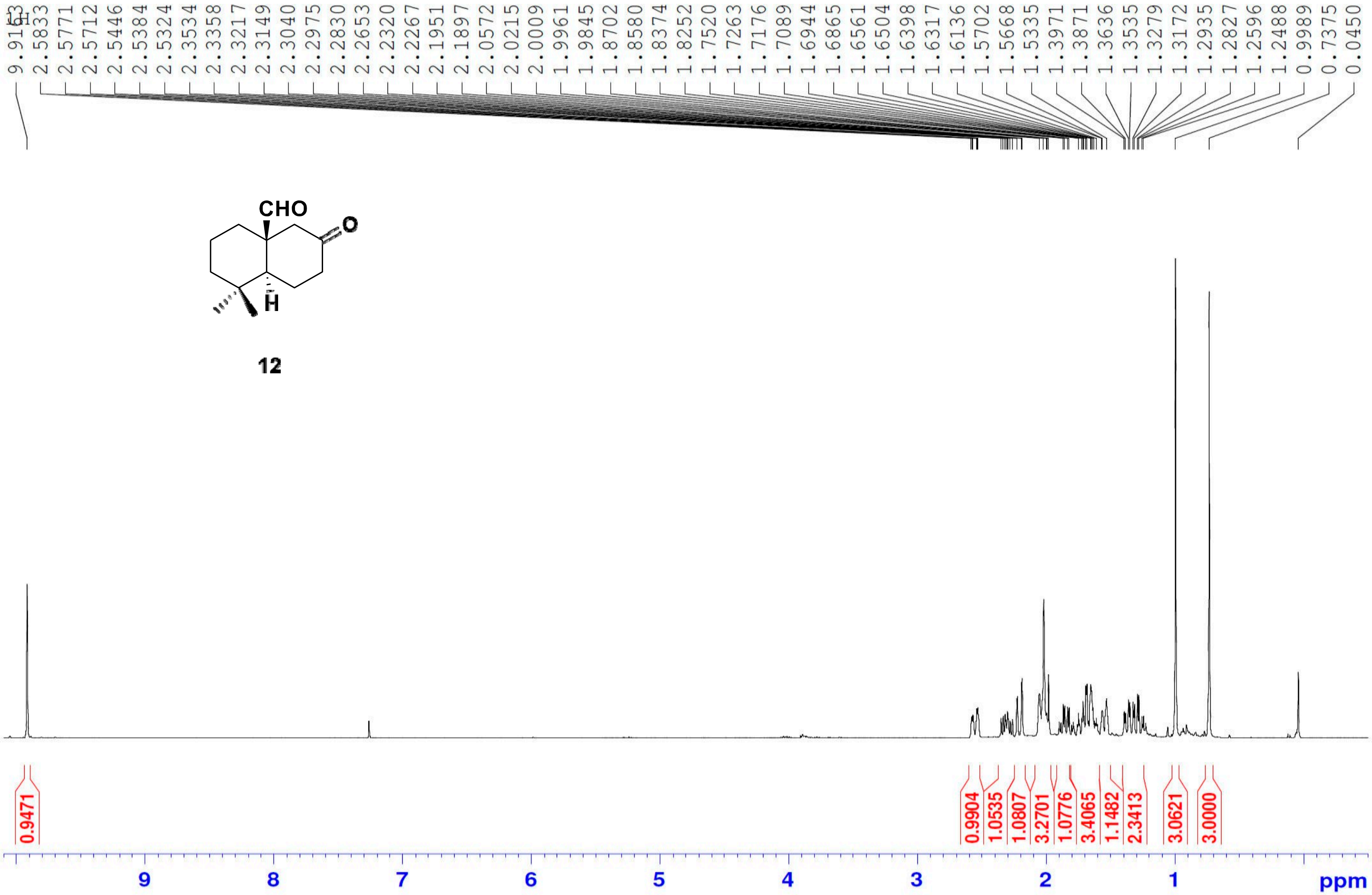

12

^{1}H NMR of compound 12 (400 MHz, $CDCl_3$)

Figure S25

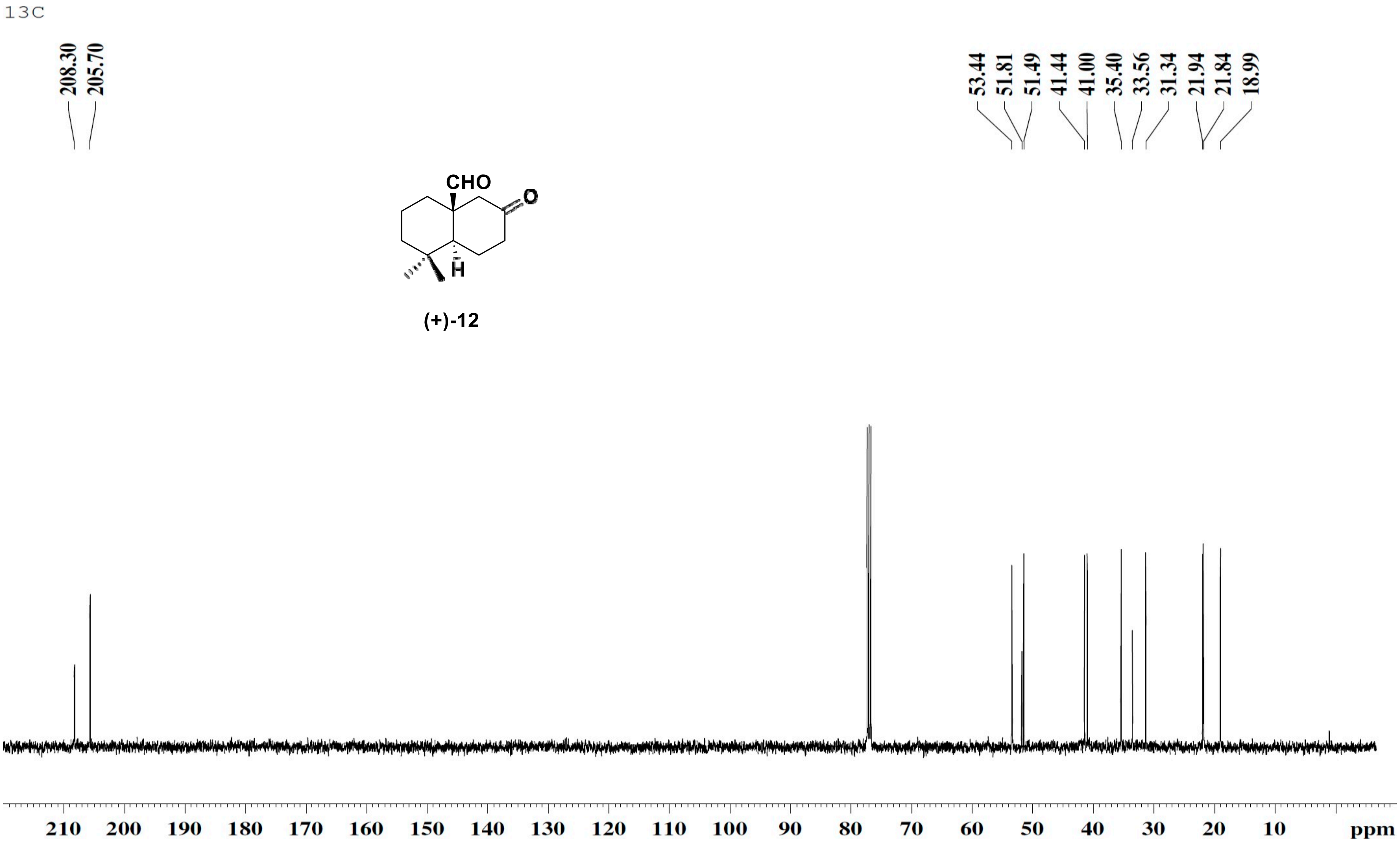

^{13}C NMR of compound (+)-**12** (100 MHz, $CDCl_3$)

Figure S26

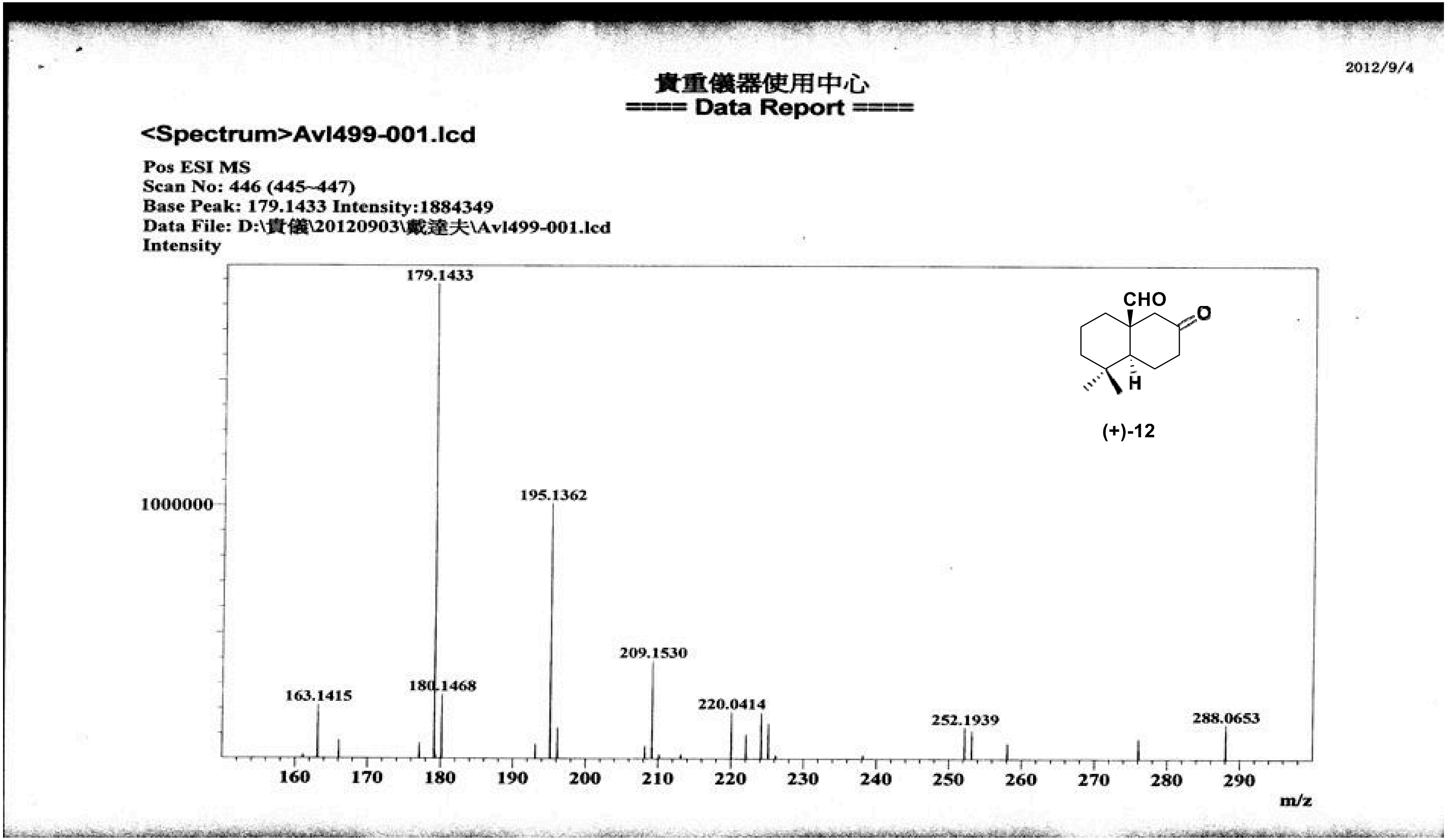

HRMS (ESI) of compound **12**

Figure S27

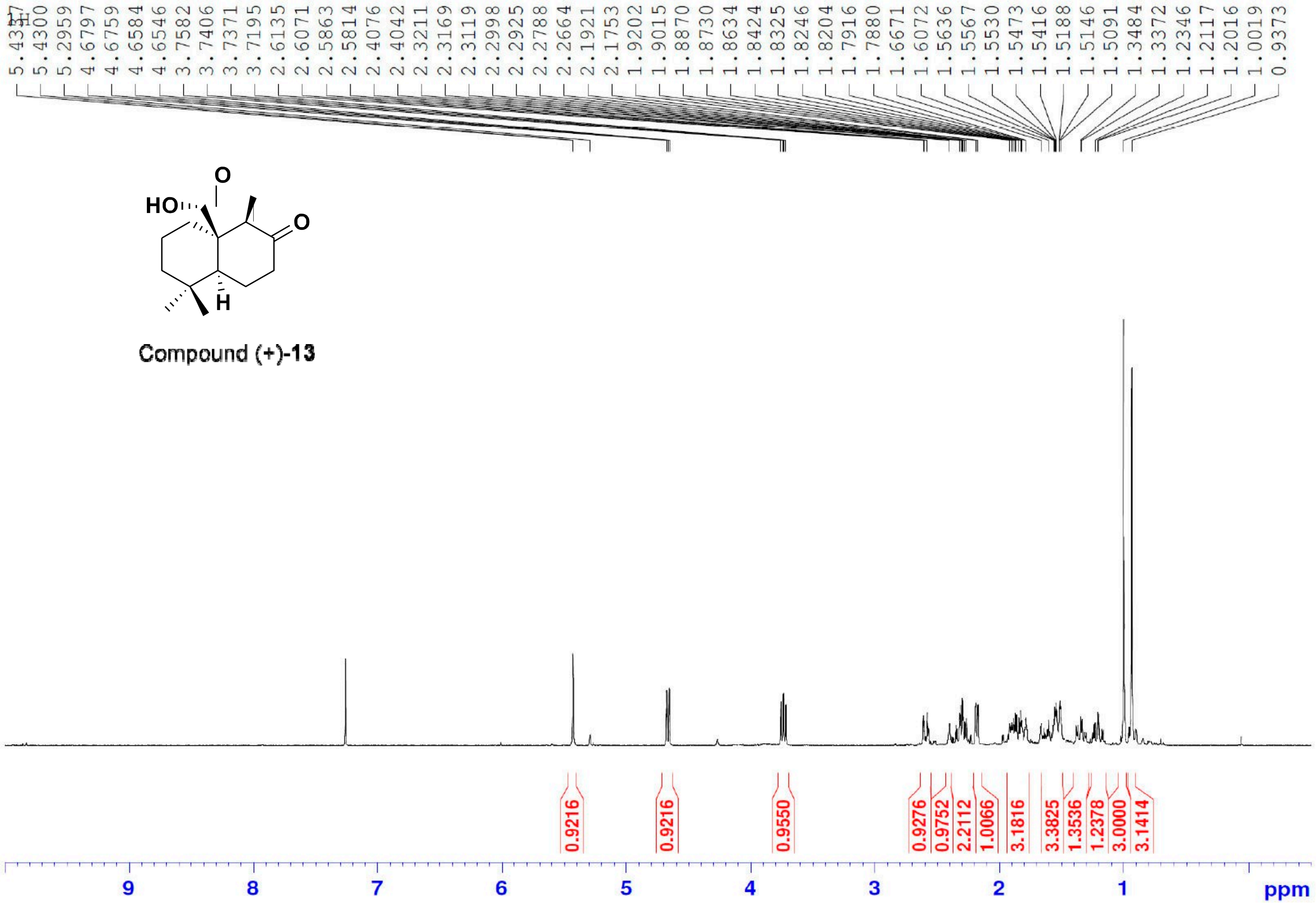

^{1}H NMR of compound (+)-**13** (400 MHz, $CDCl_3$)

Figure S28

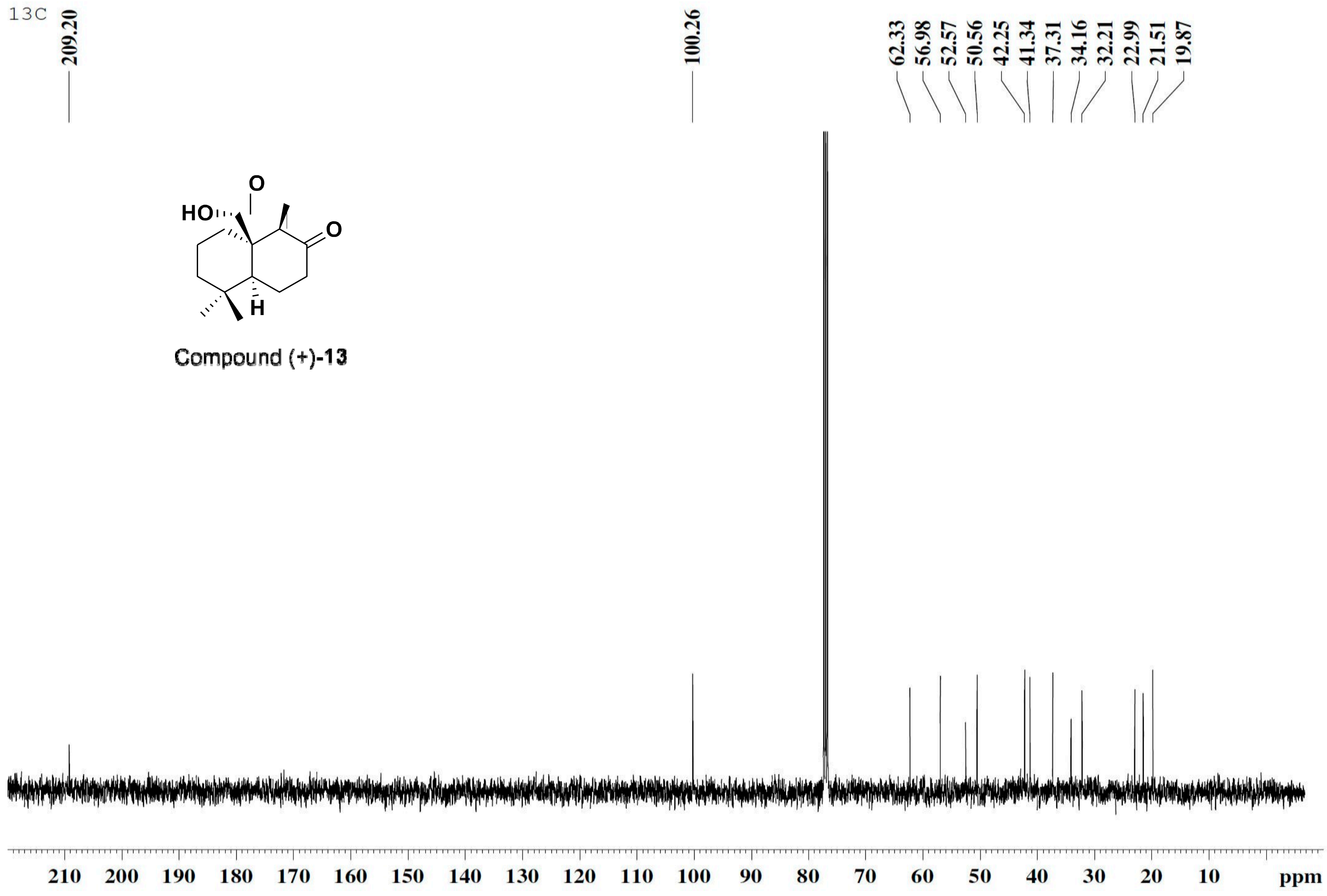

Compound (+)-13

^{13}C NMR of compound (+)-**13** (100 MHz, $CDCl_3$)

Figure S29

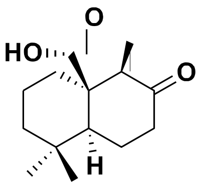

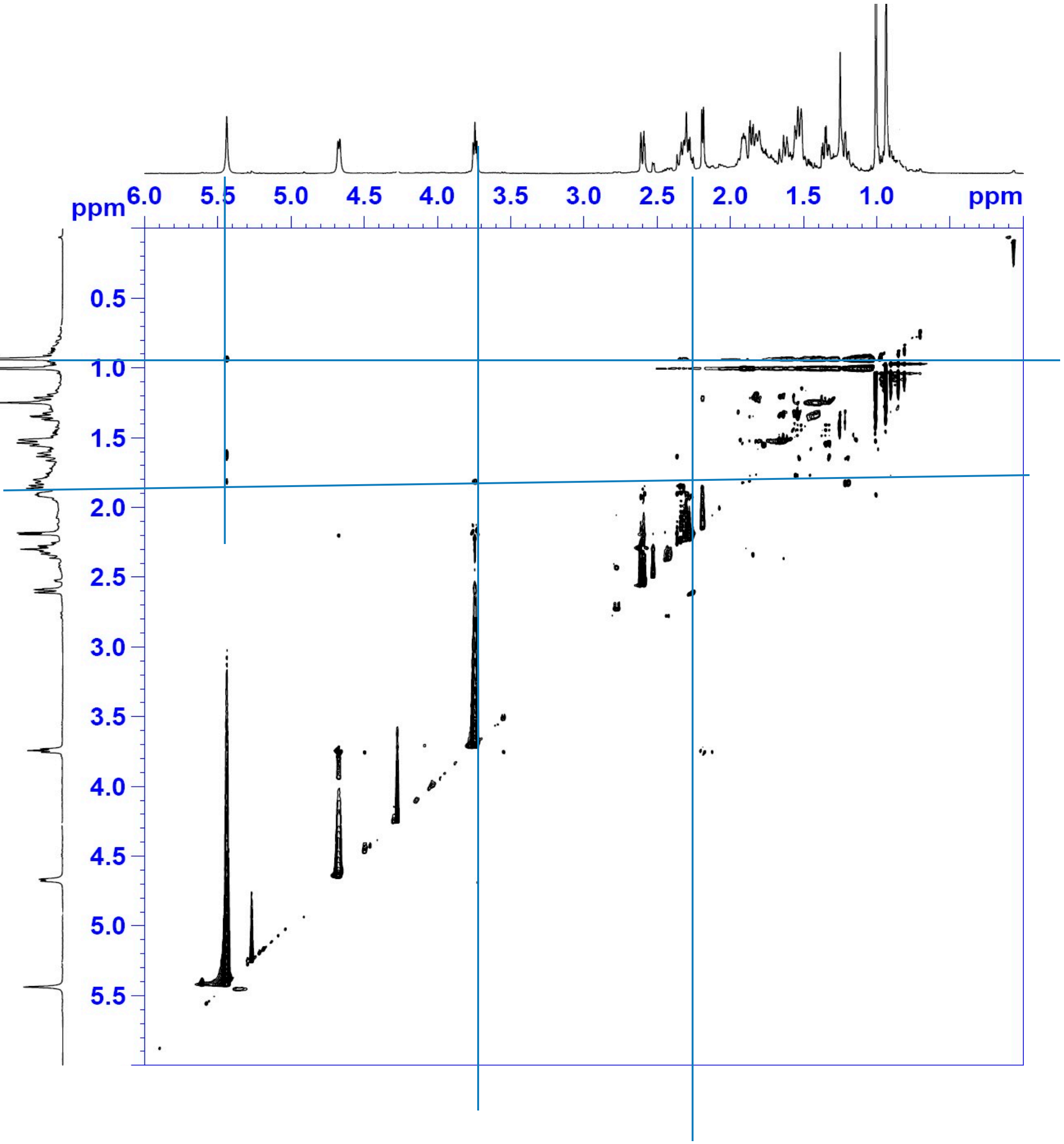

2D NOESY of compound (+)-13 (600 MHz, $CDCl_3$)

Figure 530

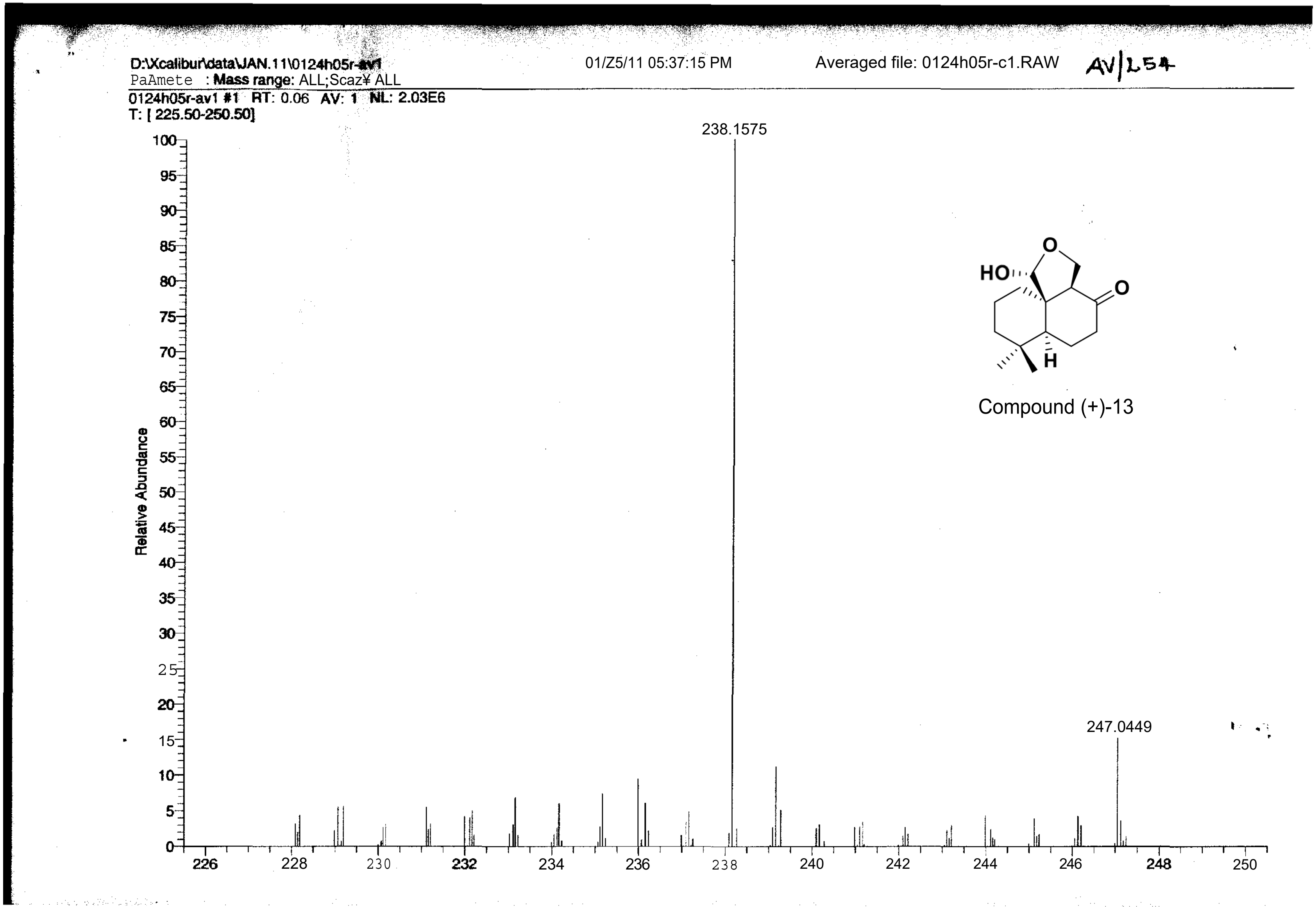

HRMS (ESI) of compound (+)-13

Figure S31

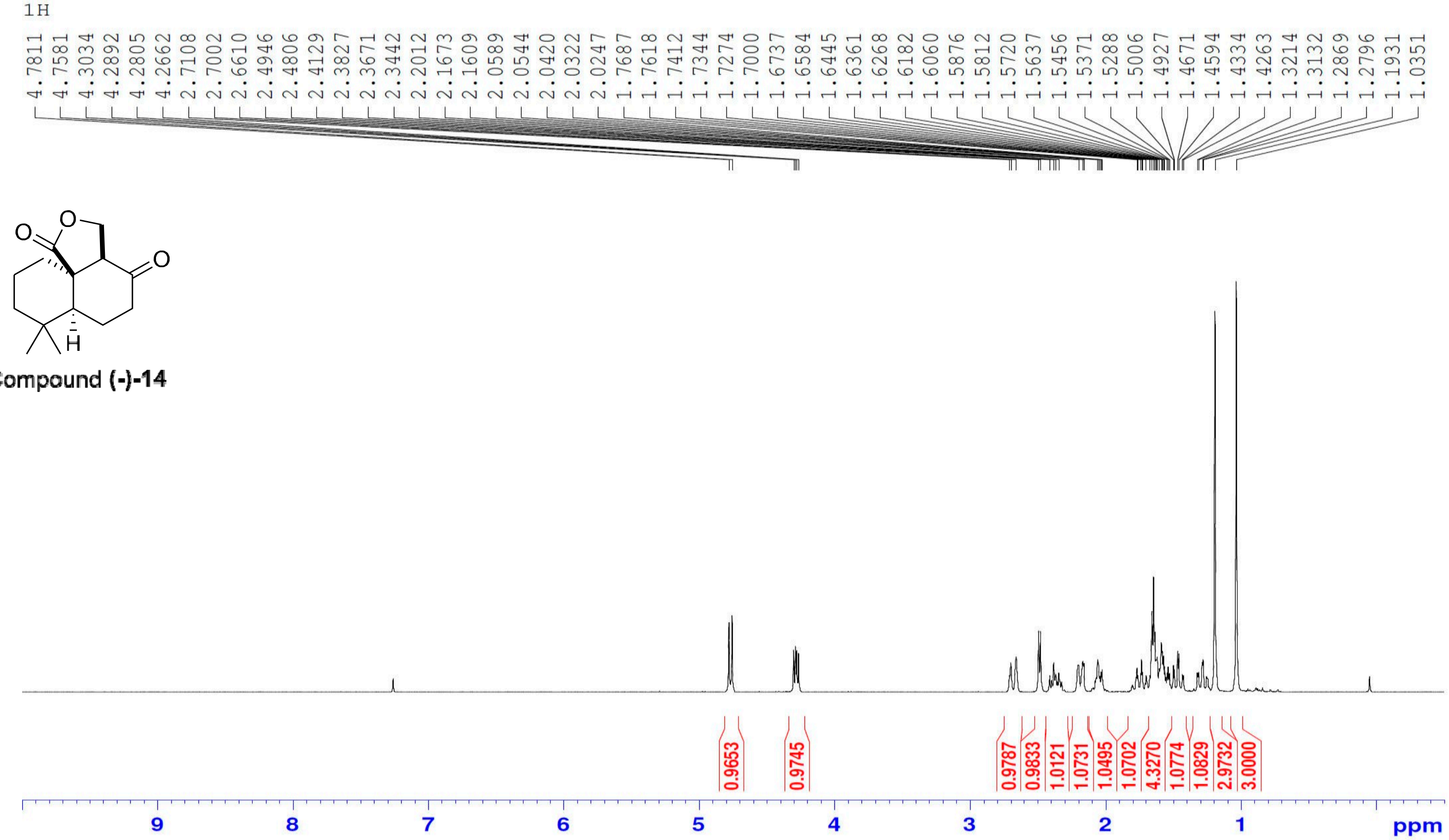

Compound (-)-14

^{1}H NMR of compound **14** (400 MHz, $CDCl_3$)

Figure S32

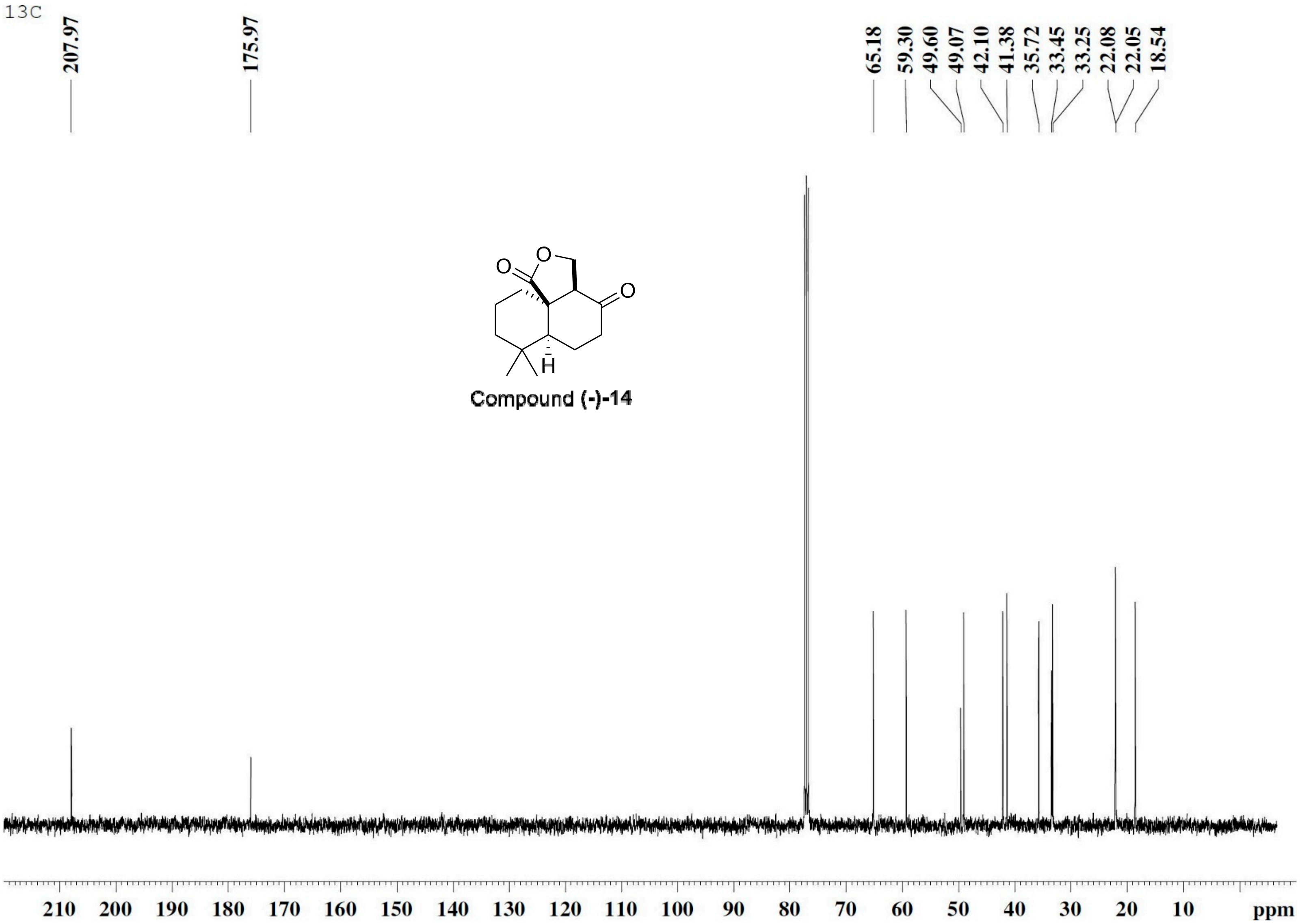

^{13}C NMR of compound **14** (100 MHz, $CDCl_3$)

Figure S33

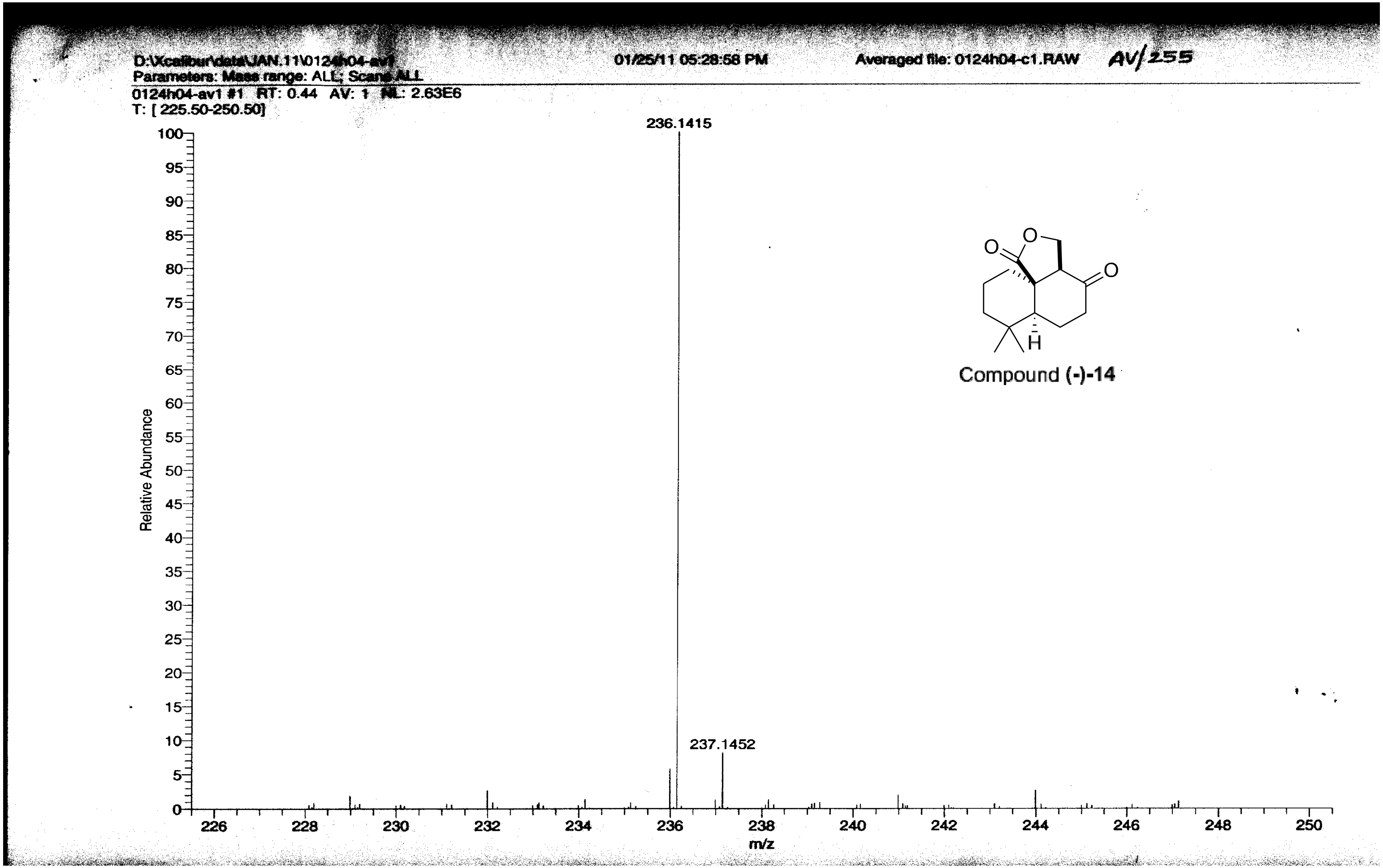

HRMS (ESI) of compound **14**

Figure S34

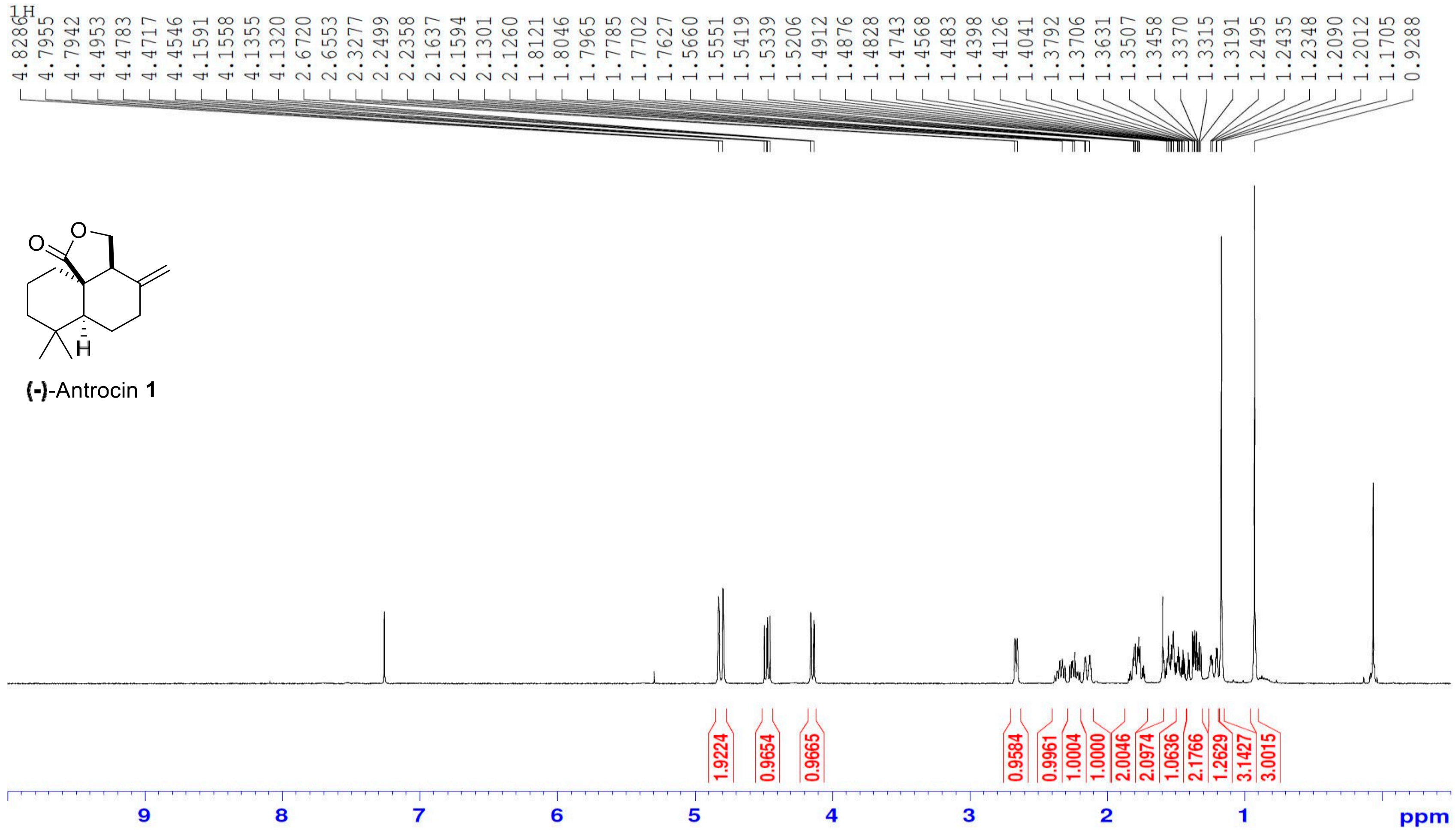

^1H NMR of (-)-antrocin **1a** (400 MHz, $CDCl_3$)

Figure S35

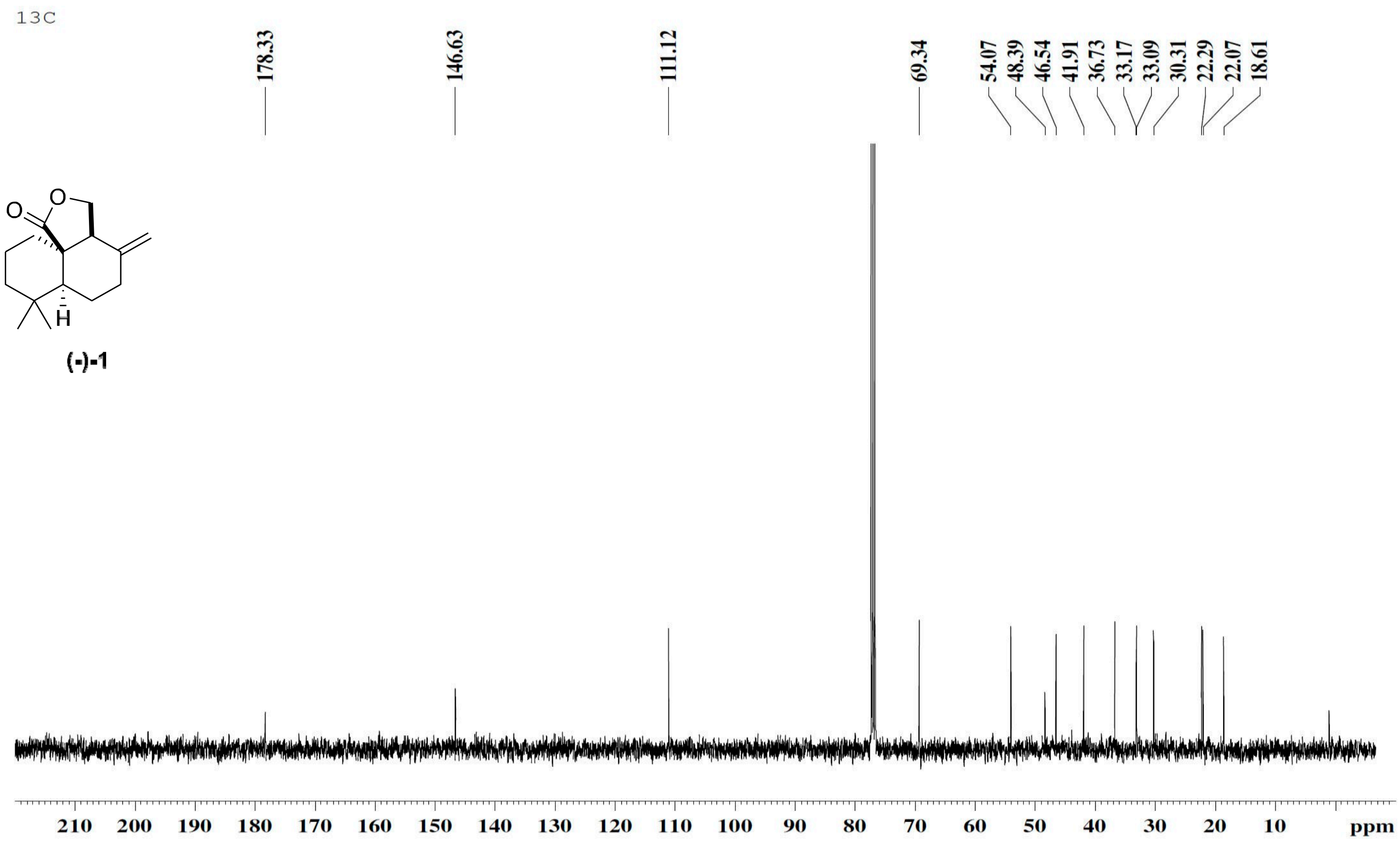

^{13}C NMR of (-)-antrocin **1**(100 MHz, $CDCl_3$)

Figure S36

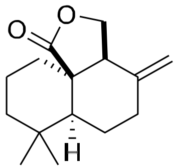

(-)-1

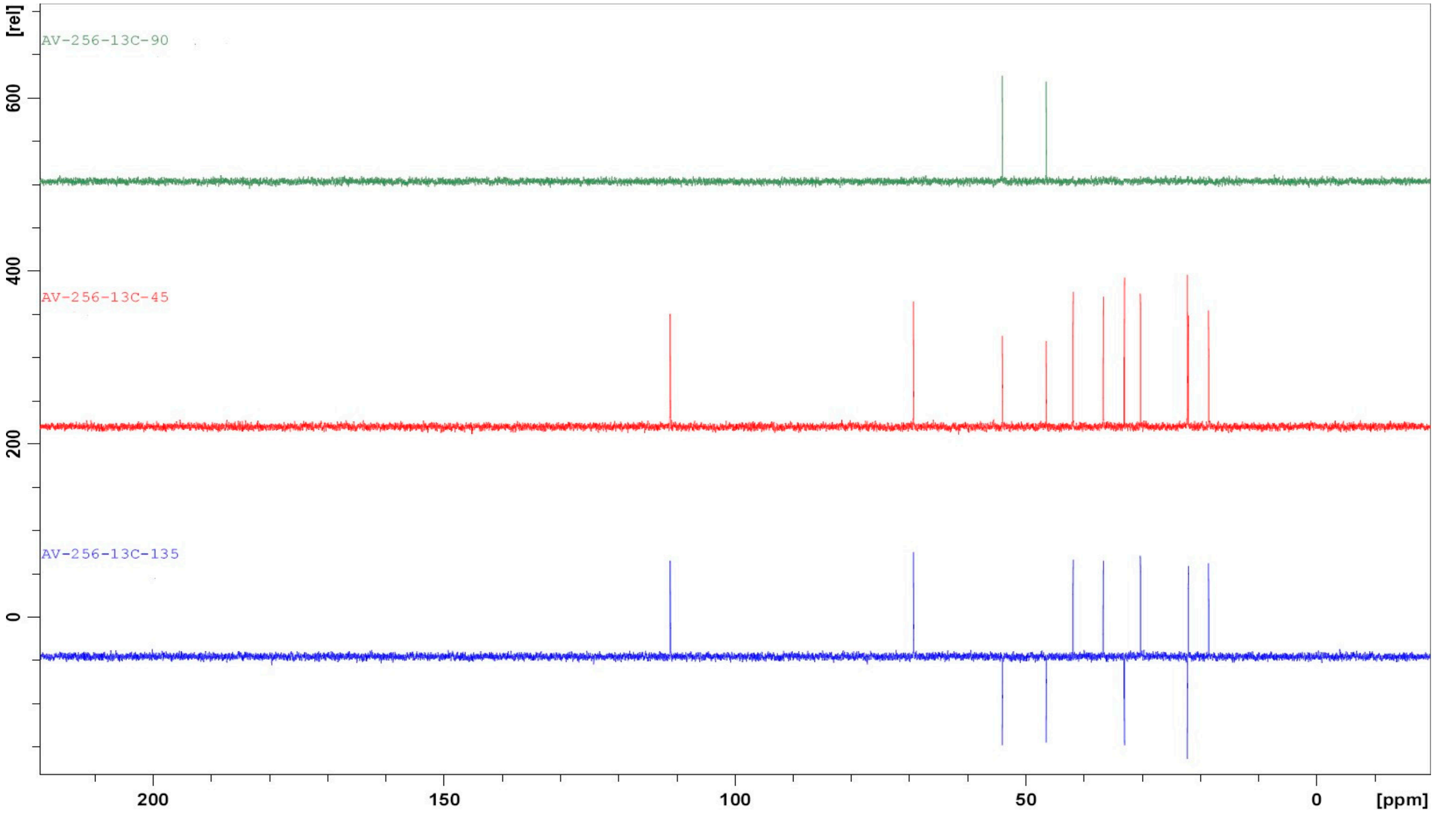

^{13}C, DEPT 135, DEPT 45 and DEPT 90 NMR Of (-)-antrocin **1** (100 MHz, $CDCl_3$)

Figure S37

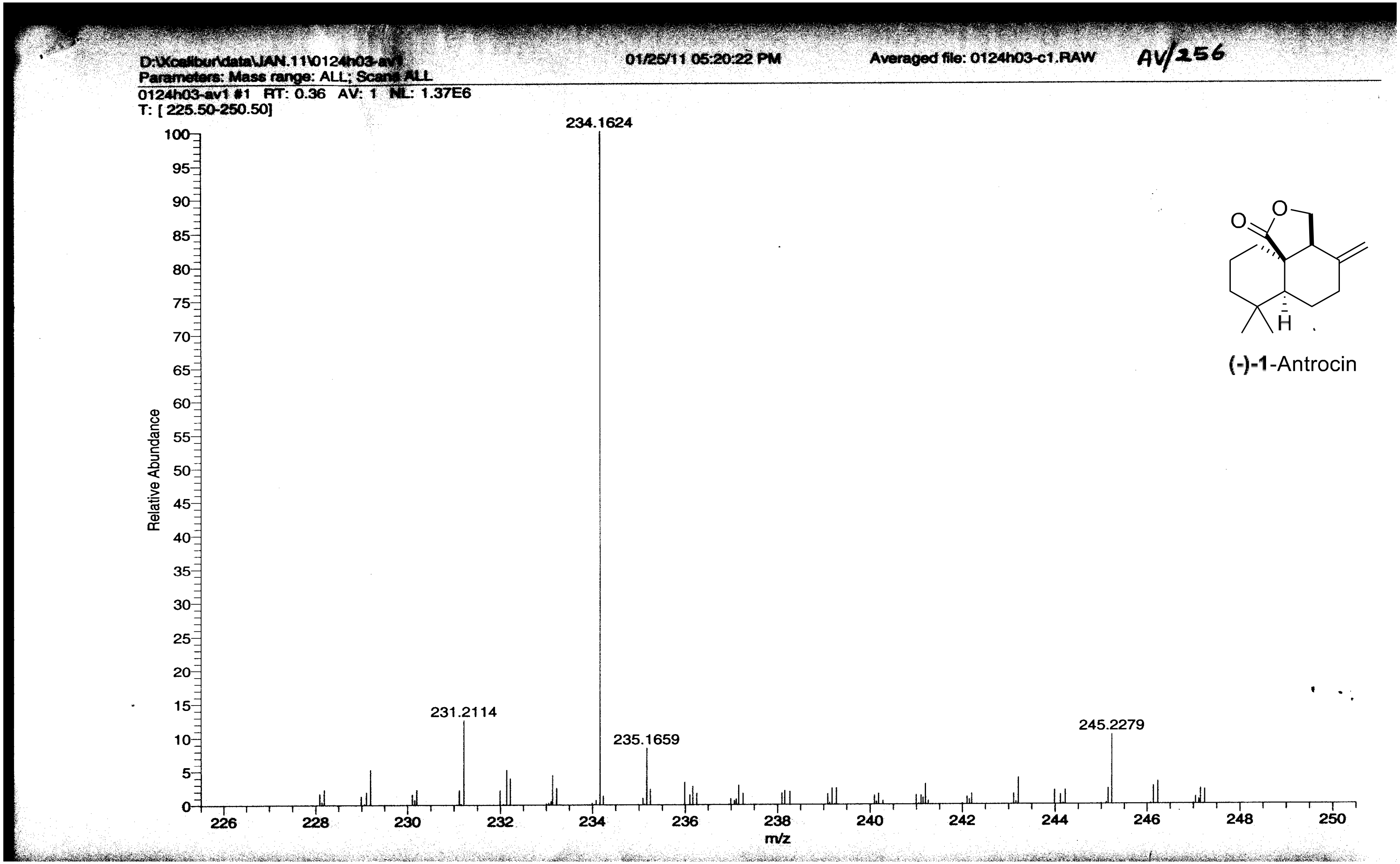

HRMS (ESI) of (-)-antrocin **1**

Figure S38

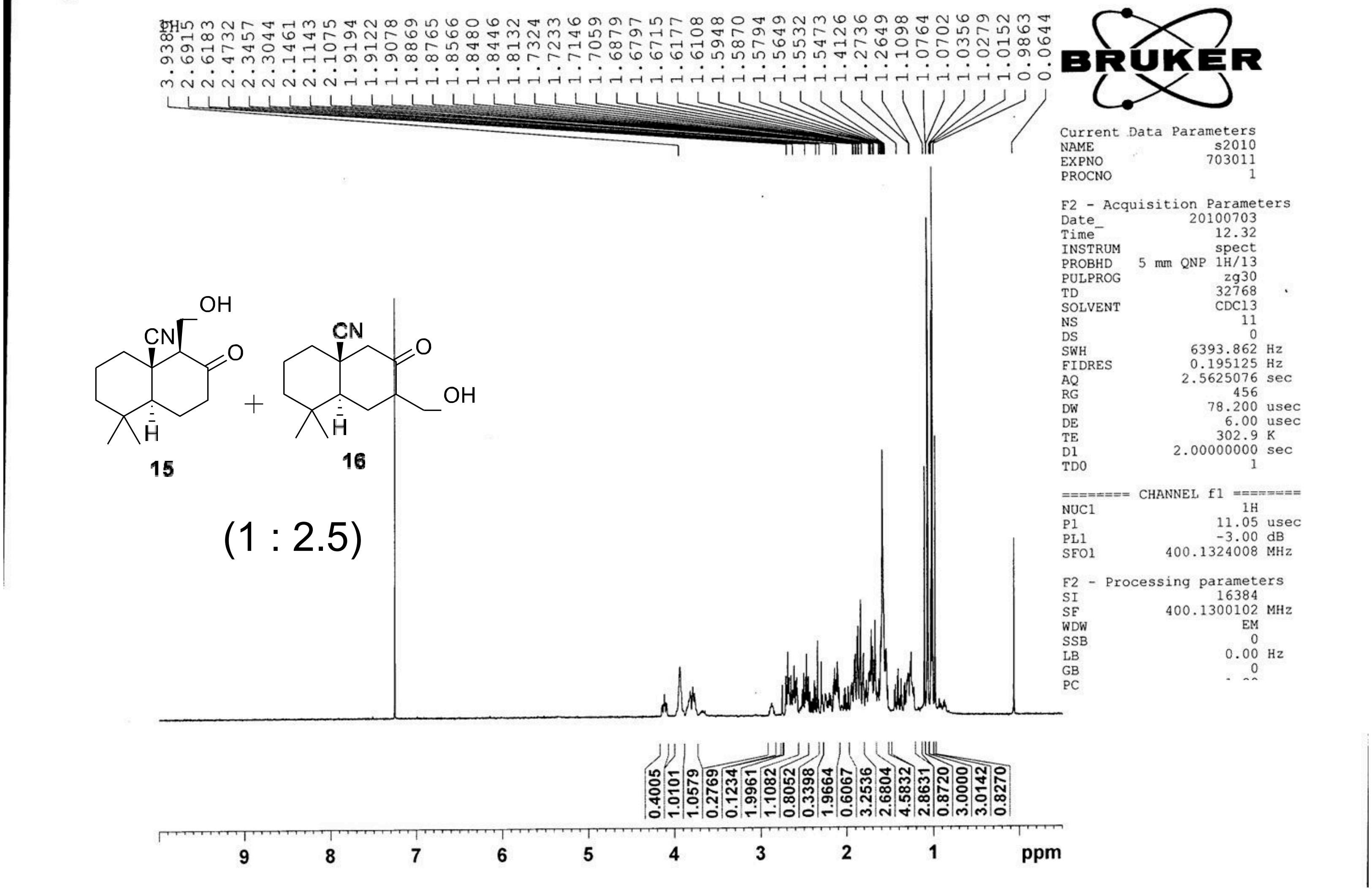

^{1}H NMR of compound 15 and 16 mixture (400 MHz, $CDCl_3$)

Figure S39

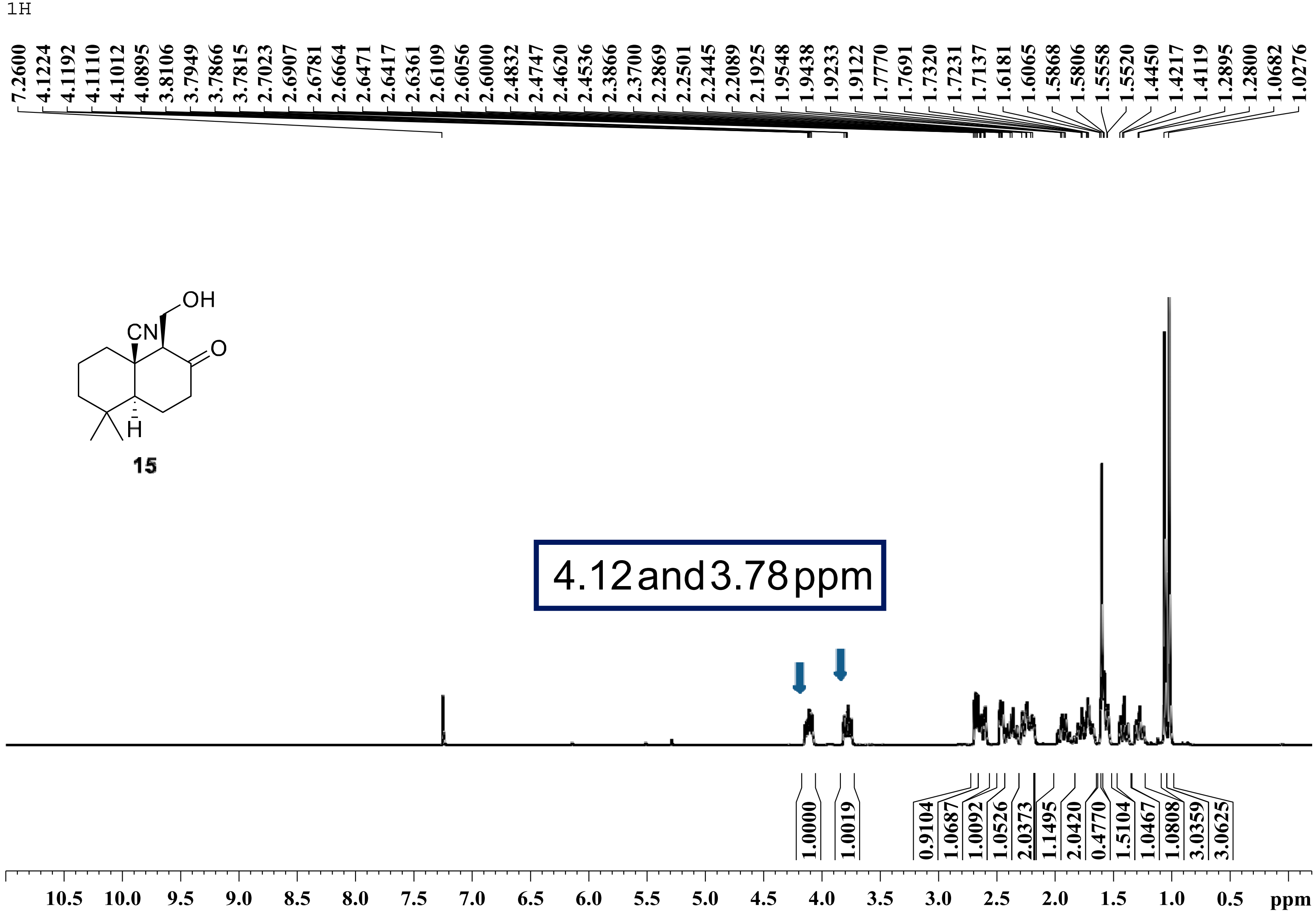

^{1}H NMR of compound **15** (400 MHz, $CDCl_3$)

Figure S40

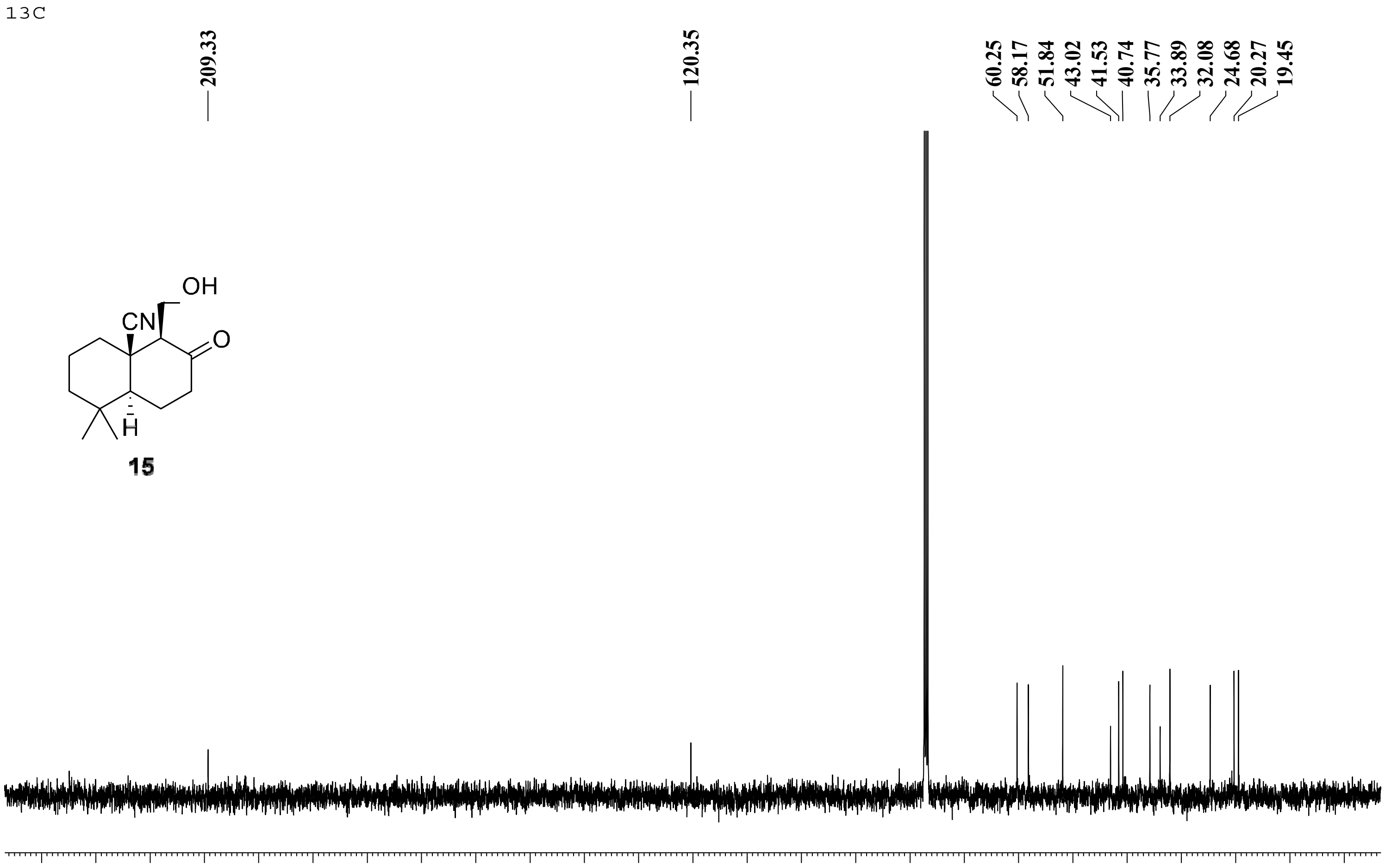

15

240 230 220 210 200 190 180 170 160 150 140 130 120 110 100 90 80 70 60 50 40 30 20 10 ppm

^{13}C NMR of compound **15** (100 MHz, $CDCl_3$)

Figure S41

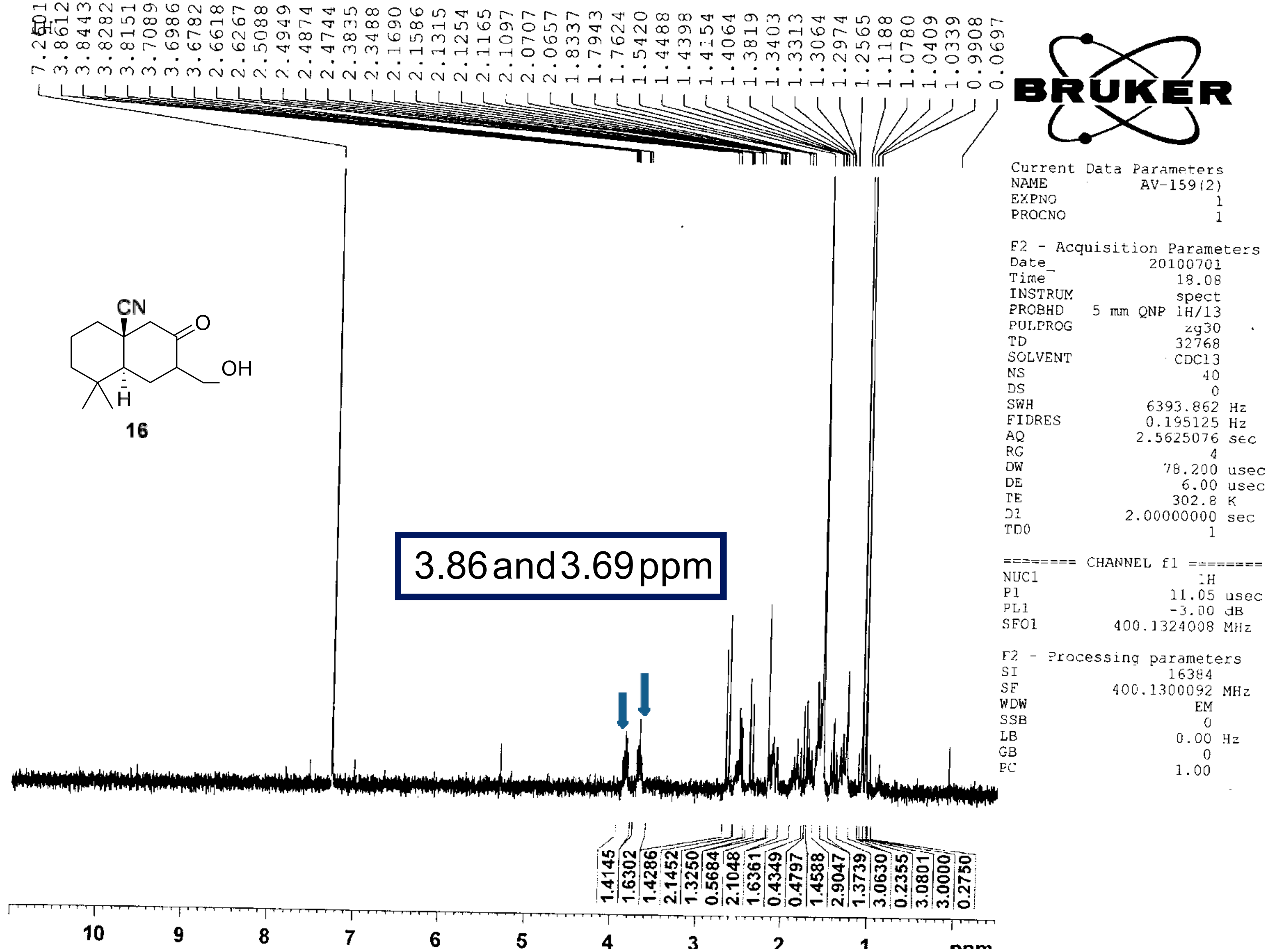

Current Data Parameters
NAME AV-159(2)
EXPNO 1
PROCNO 1

F2 - Acquisition Parameters
Date_ 20100701
Time 18.08
INSTRUM spect
PROBHD 5 mm QNP 1H/13
PULPROG zg30
TD 32768
SOLVENT CDCl3
NS 40
DS 0
SWH 6393.862 Hz
FIDRES 0.195125 Hz
AQ 2.5625076 sec
RG 4
DW 78.200 usec
DE 6.00 usec
TE 302.8 K
D1 2.00000000 sec
TD0 1

======== CHANNEL f1 ========
NUC1 1H
P1 11.05 usec
PL1 -3.00 dB
SFO1 400.1324008 MHz

F2 - Processing parameters
SI 16384
SF 400.1300092 MHz
WDW EM
SSB 0
LB 0.00 Hz
GB 0
PC 1.00

Crystallographic data for *trans* cyanobicyclic ketone, (–)-7

Table S1. Crystal data and structure refinement for *trans*-cyanobicyclicketone, **(–)7.**

Identification code	**(–)-7**
Empirical formula	$C_{13}H_{19}NO$
Formula weight	205.29
Temperature	296(2) K
Wavelength	0.626 Å
Crystal system, space group	Monoclinic
Unit cell dimensions	a = 8.7609 (7) Å α = 90°.
	b = 9.3363 (8) Å β = 90°.
	c = 14.6484 (14) Å γ = 90°.
Volume	1198.16 (18) $Å^3$
Z, Calculated density	4
No. of measured, independent and observed [$I > 2\sigma(I)$] reflections	8727, 2464, 1632
$(\sin \theta/\lambda)_{max}$ ($Å^{-1}$)	0.626
Crystal size (mm)	0.42 × 0.38 × 0.33
No. of measured, independent and observed [$I > 2\sigma(I)$] reflections	8727, 2464, 1632
R_{int}	0.046
$(\sin \theta/\lambda)_{max}$ ($Å^{-1}$)	0.626
$R[F^2 > 2\sigma(F^2)]$, $wR(F^2)$, S	0.049, 0.102, 1.02
No. of reflections	2464
No. of parameters	138
H-atom treatment	H atoms treated by a mixture of independent and constrained refinement
$\Delta\rho_{max}$, $\Delta\rho_{min}$ (e $Å^{-3}$)	0.09, −0.13

Absolute structure	Flack H D (1983), Acta Cryst. A39, 876-881
F(000)	448
Absolute structure parameter	2 (3)
Reflections	2464
Refinement method	Full-matrix least-squares on F^2

Table S2. Atomic coordinates (x 10^4) and equivalent isotropic displacement parameters ($Å^2$ x 10^3) for *trans*-cyanobicycli ketone **(–)-7**. U(eq) is defined as one third of the trace of the orthogonalized U^{ij} tensor.

	X	Y	Z	U
C(5)	2002 (2)	1074 (2)	6994 (14)	45 (5)
C(6)	1936 (2)	67.5 (19)	6224 (13)	44 (5)
C(1)	0666 (2)	316 (2)	5553 (14)	54 (5)
C(2)	893 (2)	421 (3)	6018 (16)	66 (7)
C(7)	3488 (2)	-443 (2)	5781 (15)	56 (6)
C(8)	4673 (3)	−678 (3)	6533 (16)	70 (7)
C(4)	445 (2)	1118 (2)	7480 (15)	62 (6)
C(9)	4797 (3)	539 (3)	7216 (16)	71 (7)
C(3)	854 (3)	1384 (2)	6832 (17)	63 (6)
C(10)	3276 (2)	747 (3)	7680 (14)	62 (6)
C(11)	2255 (2)	533 (3)	6621 (14)	53 (5)
N(1)	2396 (2)	3680 (2)	6370 (15)	77 (6)
O(1)	−1780 (18)	2315 (2)	6962 (15)	94 (6)
C(13)	3316 (3)	−1857 (3)	5258 (19)	84 (8)
C(12)	4050 (3)	712 (3)	5118 (16)	77 (8)

Table S3. Bond lengths [Å] and angles [°] for *trans*-cyanobicyclic ketone **(–)-7**

C(5)-C11	1.485 (3)
C(5)-C(10)	1.532 (3)
C(5)-C(4)	1.539 (3)
C(5)-C(6)	1.553 (3)
C(6)-C(1)	1.527 (3)
C(6)-C(7)	1.547 (3)
C(1)-C(2)	1.530 (3)
C(2)-C(3)	1.494 (3)
C(11)-C5-C(10)	108.37 (16)
C(11)-C(5)-C(4)	106.16 (16)
C(10)-C(5)-C(4)	110.38 (16)
C(11)-C(5)-C(6)	111.57 (16)
C(10)-C(5)-C(6)	111.51 (16)
C(4)-C(5)-C(6)	108.72 (15)
C(1)-C(6)-C(7)	115.00 (17)
C(1)-C(6)-C(5)	109.53 (15)
C(7)-C(6)-C(5)	115.31 (15)
C(6)-C(1)-C(2)	112.27 (17)
C(3)-C(2)-C(1)	111.91 (17)
C(8)-C(7)-C(12)	109.87 (19)
C(8)-C(7)-C(13)	107.63 (19)
C(7)-C(8)	1.530 (3)
C(7)-C(12)	1.532 (3)
C(7)-C(13)	1.534 (3)
C(8)-C(9)	1.517 (3)

C(4)-C(3)	1.502 (3)
C(9)-C(10)	1.509 (3)
C(3)-O(1)	1.204 (3)
C(11)-N(1)	1.139 (3)
C(12)-C(7)-C(13)	108.67 (19)
C(8)-C(7)-C(6)	109.06 (17)
C(12)-C(7)-C(6)	112.91 (17)
C(13)-C(7)-C(6)	108.56 (17)
C(9)-C(8)-C(7)	114.54 (19)
C(3)-C(4)-C(5)	112.55 (17)
C(10)-C(9)-C(8)	109.27 (18)
O(1)-C(3)-C(2)	123.0 (2)
O(1)-C(3)-C(4)	122.0 (2)
C(2)-C(3)-C(4)	114.99 (19)
C(9)-C(10)-C(5)	111.96 (17)
N(1)-C(11)-C(5)	176.3 (2)

Symmetry transformations used to generate equivalent atoms:

Table S4. Anisotropic displacement parameters ($Å^2$ x 10^3) for *trans* bicyclicketone **(–)-7**. The anisotropic displacement factor exponent takes the form: $-2\pi^2[h^2 a^{*2}U^{11} + ... + 2 h k a^* b^* U^{12}]$

	U^{11}	U^{22}	U^{33}	U^{12}	U^{13}	U^{23}
C(5)	46 (12)	41(12)	48(11)	−4 (9)	4 (10)	−1 (9)
C(6)	46 (12)	35(11)	48 (11)	−6 (9)	6 (9)	3 (9)
C(1)	54 (13)	46 (12)	61 (13)	1 (10)	−11 (11)	−5 (10)
C(2)	45 (14)	57 (14)	94 (18)	-1 (11)	−14 (12)	−2 (14)
C(7)	50 (13)	53 (13)	64 (13)	8 (11)	7 (11)	3 (12)
C(8)	45 (13)	71 (16)	95 (18)	11 (12)	3 (13)	15 (15)
C(4)	63 (15)	59 (14)	63 (14)	−4 (12)	13 (12)	−14 (11)
C(9)	54 (15)	76 (17)	84 (17)	−3 (13)	−22 (13)	17 (15)
C(3)	47 (13)	51 (14)	90 (18)	−3 (11)	13 (12)	−2 (14)
C(10)	67 (15)	63 (15)	55 (13)	−8 (12)	−14 (12)	4 (11)
C(11)	49 (13)	46 (13)	64 (13)	−6 (11)	−9 (10)	−3 (11)
N(1)	85 (16)	47 (12)	10 (15)	−9 (11)	−22 (12)	6 (13)
O(1)	64 (11)	76 (12)	14 (16)	23 (10)	20 (11)	−13 (12)
C(13)	92 (2)	68 (17)	93 (18)	21 (15)	13 (17)	−19 (15)
C(12)	71 (17)	84 (18)	76 (17)	5 (14)	27 (13)	15 (15)

Crystallographic data for ketol diastreomer, (–)-8

Table S5. Crystal data and structure refinement for ketal diastreomer **(–)-8**

Identification code	**(–)-8**
Chemical formula	$C_{19}H_{27}NO_6$
M_r	365.42
Crystal system, space group	P2(1)
Temperature (K)	296
a, *b*, *c* (Å)	12.9504 (5), 11.5452 (5), 13.3239 (5)
α, β, γ (°)	90, 96.190 (1), 90
V (Å^3)	1980.51 (14)
Z	4
Radiation type	Mo $K\alpha$
μ (mm^{-1})	0.09
Crystal size (mm)	0.49 × 0.48 × 0.25
**Data collection**	
Diffractometer	–
Absorption correction	–
No. of measured, independent and observed [$I > 2\sigma(I)$] reflections	19868, 7836, 6201
R_{int}	0.032
(sin θ/λ)$_{max}$ (Å^{-1})	0.626
**Refinement**	
$R[F^2 > 2\sigma(F^2)]$, $wR(F^2)$, S	0.046, 0.131, 1.03
No. of reflections	7836

No. of parameters	477
No. of restraints	1
H-atom treatment	H atoms treated by a mixture of independent and constrained refinement
$\Delta\rho_{max}$, $\Delta\rho_{min}$ (e Å^{-3})	0.27, −0.19
Absolute structure	Flack H D (1983), Acta Cryst. A39, 876-881
Absolute structure parameter	0.4 (9)

Table S6. Atomic coordinates (x 10^4) and equivalent isotropic displacement parameters (Å^{2}x 10^3) for ketol diastreomer **(–)-8.** U(eq) is defined as one third of the trace of the orthogonalized U^{ij} tensor.

	x	y	z	U(eq)
C(1)	8757 (19)	−2316 (2)	6866 (2)	50. (6)
C(2))	879 (2)	−2889 (2)	5843 (2)	55 (6)
C(3)	832 (2)	−2091 (2)	5008 (2)	58 (7)
C(4)	884 (19)	−904 (2)	9889 (17)	46 (5)
C(5)	889 (16)	−355 (18)	60565 (16)	36 (4)
C(6)	933 (15)	−1146 (18)	6933 (16)	35 (4)
C(7)	9198 (17)	−552 (2)	7951 (16)	42 (5)
C(8)	9647 (16)	655 (2)	8045 (15)	39 (5)
C(9)	9244 (2)	1410 (2)	7168 (17)	47 (5)
C(10)	9412 (2)	835 (19)	6162 (16)	44 (5)
C(11)	9921 (2)	–1021 (3)	4598 (2)	60 (7)
C(12)	8156 (3)	−151 (3)	4243 (2)	77 (9)
C(13)	10454 (17)	−1418 (2)	6915 (18)	43 (5)
C(14)	11569 (19)	2403 (2)	8744 (2)	48 (6)
C(15)	11184 (17)	1204 (2)	8996 (15)	42 (5)

C((16)	10286 (17)	1254 (2)	9660 (16)	44. (5)
C(170)	10345 (2)	243 (3)	10408(18)	53 (6)
C(18)	12521 (3)	3987 (3)	9491 (4)	106 (14)
C(19)	11024 (4)	−413 (3)	2004 (3)	95 (12)
C(20)	39820 (2)	7605 (2)	7045 (2)	54 (6)
C(21)	4174 (2)	6908 (2)	6118 (2)	61 (7)
C(22)	3559 (2)	7402 (2)	5184 (2)	61 (7)
C(23)	37976 (19)	8683 (2)	9763 (19)	51 (6)
C(24)	36761 (16)	9391 (2)	59474 (17)	41 (5)
C(25)	4262 (16)	8887 (2)	6932 (17)	42 (5)
C(26)	3957 (17)	9574 (2)	7847 (18)	46 (5)
C(27)	4108 (17)	10861(2)	7762 (17)	45 (5)
C(28)	3551 (19)	11330 (2)	6793 (17)	49 (5)
C(29)	3889 (19)	10692 (2)	5884 (18)	47 (5)
C(30)	4867 (2)	8798 (3)	4592 (2)	70 (8)
C(31)	2976 (3)	9108 (3)	4135 (2)	70 (8)
C(32)	5409 (17)	8957 (2)	6943 (2)	52 (6)
C(33)	4450 (2)	10826 (3)	10284 (2)	64 (7)
C(34)	4603 (2)	11591 (2)	9366 (19)	53 (6)
C(35	5547 (18)	11224 (2)	8839 (17)	50 (6)
C(36_	6448 (2)	12056 (3)	9011 (2)	55 (7)
C(37)	3737 (4)	10792 (5)	11812 (3)	114(16)
C(38)	7657 (3)	12791 (4)	10301 (3)	89 (11)
N(1)	11288 (17)	−1701 (2)	6934 (2)	69(7)
N(2)	6287 (18)	8958 (3)	7000 (2)	85 (8)
O(1)	10764 (12)	5894 (15)	8141 (11)	46 (4)

O(2)	9393 (11)	11883 (16)	8962 (11)	49 (4)
O(3)	12059 (15)	28564 (18)	9578 (15)	67 (5)
O(4)	11466 (17)	28518 (19)	7937 (16)	71 (6)
O(5)	10914 (2)	482 (2)	11237 (15)	87 (7)
O(6)	9979 (3)	−707 (2)	10259 (17)	103 (9)
O(7)	5167 (12)	11197 (16)	78095 (11)	48 (4)
O(8)	3741 (13)	11436 (17)	86203 (12)	56 (5)
O(9)	4017 (2)	11411 (3)	10951 (17)	93 (8)
O(10)	4698 (3)	9844 (3)	10366 (2)	125 (11)
O(11)	6795 (16)	12034 (2)	9989 (15)	74 (6)
O(12)	6796 (18)	12641 (2)	8398 (17)	80 (6)

Table S7. Bond lengths [Å] and angles [°] for ketol diastreomer **(–)-8**

C(1)-C(2)	1.522 (4)
C(1)-C(6)	1.538 (3)
C(2)-C(3)	1.522 (4)
C(3)-C(4)	1.531 (4)
C(4)-C(12)	1.534 (4)
C(4)-C(11)	1.544 (4)
C(4)-C(5)	1.552 (3)
C(5)-C(10)	1.529 (3)
C(5)-C(6)	1.541 (3)
C(6)-C(13)	1.491 (3)
C(6)-C(7)	1.545 (3)

C(7)-C(8)	1.511 (3)
C(8)-O(2)	1.438 (2)
C(8)-O(1)	1.440 (3)
C(8)-C(9)	1.505 (3)
C(9)-C(10)	1.532 (3)
C(13)-N(1)	1.127 (3)
C(14)-O(4)	1.187 (3)
C(14)-O(3)	1.327 (3)
C(14)-C(15)	1.521 (4)
C(15)-O(1)	1.401 (3)
C(15)-C(16)	1.537 (3)
C(16)-O(2)	1.405 (3)
C1(6)-C(17)	1.531 (4)
C(17)-O(6)	1.203 (4)
C(17)-O(5)	1.290 (3)
C(18)-O(3)	1.445 (4)
C(19)-O(5)	1.449 (4)
C(2)-C(1)-C(6)	111.75 (19)
C(3)-C(2)-C(1)	109.9 (2)
C(2)-C(3)-C(4)	114.1 (2)
C(3)-C(4)-C(12)	106.9 (2)
C(3)-C(4)-C(11)	110.1 (2)
C(12)-C(4)-C(11)	108.4 (2)
C(3)-C(4)-C(5)	109.0 (2)
C(12)-C(4)-C(5)	109.1 (2)

C(11)-C(4)-C(5)	113.23 (19)
C(10)-C(5)-C(6)	110.09 (17)
C(10)-C(5)-C(40	115.22 (18)
C(6)-C(5)-C(4)	115.44 (17)
C(13)-C(6)-C(1)	106.26 (18)
C(13)-C(6)-C(5)	112.87 (17)
C(1)-C(6)-C(5)	109.99 (18)
C(13)-C(6)-C(7)	107.91 (18)
C(1)-C(6)-C(7)	110.06 (17)
C(5)-C(6)-C(7)	109.67 (17)
C(8)-C(7)-C(6)	113.87 (17)
O(2)-C(8)-O(1)	105.43 (16)
O(2)-C(8)-C(9)	108.67 (18)
O(1)-C(8)-C(9)	111.20 (18)
O(2)-C(8)-C(7)	110.12 (17)
O(1)-C(8)-C(7)	109.43 (17)
C(9)-C(8)-C(7)	111.80 (18)
C(8)-C(9)-C(10)	111.00 (18)
C(9)-C(10)-C(5)	111.59 (18)
N(1)-C(13)-C(6)	174.8 (3)
O(4)-C(14)-O(30	125.7 (2)
O(4)-C(14)-C(15)	126.1 (2)
O(3)-C(14)-C(15)	108.1 (2)
O(1)-C(15)-C(14)	112.90 (19)
O(1)-C(15)-C(16)	103.59 (17)
C(14)-C(15)-C(16)	112.33 (19)

O(2)-C(16)-C(17)	111.6 (2)
O(2)-C(16)-C(15)	103.68 (17)
C(17)-C(16)-C(15)	111.0 (2)
O(6)-C(17)-O(5)	121.0 (3)
O(6)-C(17)-C(16)	126.7 (2)
O(5)-C(17)-C(16)	112.2 (2)
C(21)-C(20)-C(25)	111.8 (2)
C(22)-C(21)-C(20)	110.6 (2)
C(21)-C(22)-C(23)	114.3 (2)
C(30)-C(23)-C(31)	107.9 (2)
C(20)-C(21)	1.517 (4)
C(20)-C(25)	1.535 (4)
C(21)-C(22)	1.515 (4)
C(22)-C(23)	1.541 (4)
C(23)-C(30)	1.533 (4)
C(23)-C(31)	1.540 (4)
C(23)-C(24)	1.553 (3)
C(24)-C(29)	1.532 (3)
C(24)-C(25)	1.556 (3)
C(25)-C(32)	1.485 (3)
C(25)-C(26)	1.542 (3)
C(26)-C(27)	1.514 (4)
C(27)-O(7)	1.418 (3)
C((27)-O(8)	1.441 (3)
C(27)-C(28)	1.510 (3)
C(28)-C(29)	1.526 (3)

C(32)-N(2)	1.131 (3)
C(33)-O(10)	1.181 (4)
C(33)-O(9)	1.293 (4)
C(33)-C(34)	1.538 (4)
C(34)-O(8)	1.424 (3)
C(34)-C(35)	1.534 (3)
C(35)-O(7)	1.407 (3)
C(35)-C(36)	1.509 (4)
C(36)-O(12)	1.187 (3)
C(36)-O(11)	1.333 (3)
C(37)-O(9)	1.433 (4)
C(38)-O(11)	1.443 (4)

C(30)-C(23)-C(22)	110.4 (2)
C(31)-C(23)-C(22)	107.5 (2)
C(30)-C(23)-C(24)	114.1 (2)
C(31)-C(23)-C(24)	108.4 (2)
C(22)-C(23)-C(24)	108.4 (2)
C(29)-C(24)-C(23)	115.81 (19)
C(29)-C(24)-C(25)	109.79 (19)
C(23)-C(24)-C(25)	114.94 (18)
C(32)-C(25)-C(20)	107.37 (19)
C(32)-C(25)-C(26)	107.8 (2)
C(20)-C(25)-C(26)	109.58 (19)
C(32)-C(25)-C(24)	112.60 (19)
C(20)-C(25)-C(24)	110.1 (2)

C(26)-C(25)-C(24)	109.35 (17)
C2)(7)-C(26)-C(25)	113.78 (19)
O(7)-C(27)-O(8)	104.13 (18)
O(7)-C(27)-C(28)	108.20 (19)
O(8)-C(27)-C(28)	110.31 (18)
O(7)-C(7)-C(26)	113.11 (18)
O(8)-C(27)-C(26)	109.48 (19)
C(28)-C(27)-C(26)	111.4 (2)
C(27)-C(28)-C(29)	110.65 (19)
C(28)-C(29)-C(24)	111.50 (19)
N(2)-C(32)-C(25)	175.5 (3)
O(10)-C(33)-O(9)	125.1 (3)
O(10)-C(33)-C(34)	124.6 (3)
O(9)-C(33)-C(34)	110.3 (3)
O(8)-C(34)-C(35)	104.44 (19)
O(8)-C(34)-C(33)	108.9 (2)
C(35)-C(34)-C(33)	112.5 (2)
O(7)-C(35)-C(36)	110.2 (2)
O(7)-C(35)-C(34)	103.91 (18)
C(36)-C(35)-C(34)	113.3 (2)
O(12)-C(36)-O(11)	124.8 (3)
O(12)-C(36)-C(35)	127.1 (2)
O(11)-C(36)-C(35)	108.1 (2)
C(15)-O(1)-C(80)	110.15 (15)
C(16)-O(2)-C(8)	109.78 (16)
C(14)-O(3)-C(18)	116.9 (3)

C(17)-O(5)-C(19)	117.1 (3)
C(35)-O(7)-C(27)	106.47 (16)
C(34)-O(8)-C(27)	108.03 (18)
C(33)-O(9)-C(37)	116.9 (3)
C(36)-O(11)-C(38)	116.1 (3)

Symmetry transformations used to generate equivalent atoms:
#1 -x,y-1/2,-z+2 #2 x-1,y,z #3 x,y,z-1 #4 -x,y+1/2,-z+2
#5 x+1, y,z

Table S8. Anisotropic displacement parameters ($Å^2$ x 10^3) for ketol diastreomer, **(–)-8**. The anisotropic displacement factor exponent takes the form: $-2\pi^2$[$h^2 a^{*2} U^{11}$ + ... + 2 h k a* b* U^{12}]

	U^{11}	U^{22}	U^{33}	U^{12}	U^{13}	U^{23}
C(1)	45 (12)	39 (13)	68 (16)	−5 (10)	11 (11)	5 (12)
C(2)	52 (14)	32 (13)	82 (18)	−8 (10)	5 (13)	−13 (12)
C(3)	55 (14)	51 (15)	64 (16)	−3 (12)	−5 (12)	−21 (13)
C(4)	54 (13)	40 (13)	43 (12)	4 (10)	−2 (10)	−10 (10)
C(5)	34 (10)	34 (11)	41 (11)	3 (8)	2 (8)	−11 (9)
C(6)	30 (9)	32 (10)	44 (11)	2 (8)	4 (8)	3 (9)
C(7)	39 (11)	47 (13)	40 (12)	3 (9)	7 (9)	5 (10)
C(8)	44 (11)	43 (12)	30 (10)	3 (9)	5 (8)	−6 (9)
C(9)	64 (14)	33 (12)	44 (12)	4 (10)	3 (10)	−6 (10)
C(10)	65 (14)	32 (11)	34 (11)	−1 (10)	4 (10)	2 (9)
C(11)	83 (19)	49 (15)	52 (15)	−0.000 (13)	4 (14)	−9 (12)
C(12)	107 (2)	68 (2)	51 (16)	2 (18)	−4 (16)	−11 (14)
C(13)	34 (11)	40 (12)	54 (13)	1 (9)	3 (9)	−12 (10)

C(14)	44 (12)	46 (13)	54 (15)	1 (10)	5 (11)	−6 (12)
C(15)	45 (11)	42 (12)	38 (11)	3 (10)	2 (9)	−5 (10)
C(16)	48 (12)	49 (13)	36 (11)	1 (11)	2 (9)	−6 (10)
C(17)	56 (14)	65 (17)	37 (12)	−3 (12)	4 (10)	−2 (12)
C(18)	90 (3)	68 (2)	15 (4)	−3 (2)	5 (3)	−37 (3)
C(19)	13 (3)	83 (2)	60 (19)	−2 (2)	−2 (2)	23 (18)
C(20)	46 (13)	44 (14)	71 (16)	3 (10)	5 (12)	12 (12)
C(21)	54 (15)	41 (14)	8 (2)	2 (11)	8 (14)	−6 (14)
C(22)	58 (15)	54 (15)	71 (18)	1 (12)	5 (13)	−11 (13)
C(23)	51 (14)	50 (15)	54 (14)	3 (11)	1 (11)	−2 (11)
C(24)	31 (10)	44 (13)	50 (13)	0 (9)	8 (9)	4 (10)
C(25)	30 (10)	45 (12)	52 (13)	1 (9)	6 (9)	8 (11)
C(26)	36 (10)	56 (15)	45 (13)	5 (10)	5 (9)	10 (11)
C(27)	37 (11)	55 (15)	45 (12)	2 (10)	7 (9)	1 (10)
C(28)	54 (13)	45 (13)	49 (13)	2 (11)	1 (10)	2 (11)
C(29)	51 (13)	44 (13)	46 (12)	3 (10)	7 (10)	9 (10)
C(30)	71 (18)	70 (19)	75 (19)	4 (15)	4 (16)	−6 (16)
C(31)	79 (2)	75 (2)	54 (16)	9 (15)	−2 (14)	−8 (14)
C(32)	34 (12)	49 (14)	72 (16)	6 (10)	5 (11)	−.2 (12)
C(33)	63 (16)	79 (2)	50 (15)	0 (15)	5 (12)	−1 (14)
C(34)	51 (13)	61 (17)	48 (14)	1 (11)	8 (11)	−8 (12)
C(35)	49 (13)	53 (14)	49 (13)	5 (11)	4 (10)	3 (12)
C(36)	47 (13)	64 (18)	55 (16)	0 (12)	4 (12)	−8 (13)
C(37)	13 (3)	141 (4)	7 (2)	−4 (3)	4 (2)	−5 (2)
C(38)	58 (18)	118 (3)	9 (2)	−1(19)	1 (16)	−41 (2)
N(1)	42 (12)	73 (16)	91 (18)	1 (11)	5 (11)	−9 (14)

N(2)	38 (12)	92 (2)	12 (2)	1 (12)	10 (13)	−3 (18)
O(1)	41 (8)	53 (10)	40 (9)	−4 (7)	9 (7)	−15 (7)
O(2)	45 (8)	67 (11)	39 (8)	8 (8)	4 (6)	−13 (8)
O(3)	65 (12)	60 (12)	72 (13)	−1 (10)	2 (10)	−23 (10)
O(4)	81 (14)	64 (12)	66 (13)	−2 (11)	6 (10)	14 (11)
O(5)	128 (2)	68 (14)	57 (12)	−3 (13)	−30 (12)	13 (11)
O(6)	159 (3)	82 (17)	62 (14)	−5 (17)	−20 (14)	15 (12)
O(7)	44 (8)	56 (10)	41 (8)	2 (8)	5 (6)	−3 (8)
O(8)	45 (9)	74 (12)	49 (9)	2 (8)	7 (7)	−8 (9)
O(9)	98 (17)	121 (2)	65 (14)	−5 (15)	30 (12)	−7 (14)
O(10)	186 (3)	105 (2)	9 (2)	2 (2)	5 (2)	31 (17)
O(11)	62 (12)	99 (17)	60 (12)	−1.0 (11)	−2 (9)	−8 (11)
O(12)	76 (14)	90 (16)	73 (14)	−3 (12)	6 (11)	5 (12)

Crystallographic data for lactone (–)-14

Table S9. Crystal data and structure refinement for compound **(-)-14 .**

Identification code	**(-)-14**	
Empirical formula	C14 H20 O3	
Formula weight	236.30	
Temperature	150(2) K	
Wavelength	0.71073 Å	
Crystal system	Monoclinic	
Space group	P2(1)/n	
Unit cell dimensions	a = 6.2642(2) Å	□= 90°.
	b = 20.3792(8) Å	□= 0.100(15)°.
	c = 9.5788(3) Å	□ = 90°.
Volume	1222.82(7) $Å^3$	
Z	4	
Density (calculated)	1.284 Mg/m^3	
Absorption coefficient	0.089 mm^{-1}	
F(000)	512	
Crystal size	0.20 x 0.15 x 0.10 mm^3	
Theta range for data collection	2.92 to 27.50°.	
Index ranges	-7<=h<=8, -25<=k<=25, -12<=l<=12	
Reflections collected	12454	
Independent reflections	2775 [R(int) = 0.0389]	
Completeness to theta = 27.50°	98.7 %	
Absorption correction	Semi-empirical from equivalents	
Max. and min. transmission	1.00000 and 0.96063	
Refinement method	Full-matrix least-squares on F^2	
Data / restraints / parameters	2775 / 0 / 155	
Goodness-of-fit on F^2	5.709	
Final R indices [I>2sigma(I)]	R1 = 0.2837, wR2 = 0.6672	
R indices (all data)	R1 = 0.2964, wR2 = 0.6699	
Extinction coefficient	0.35(12)	
Largest diff. peak and hole	1.462 and -0.955 e.$Å^{-3}$	

Table S10. Atomic coordinates (x 10^4) and equivalent isotropic displacement

parameters (Å^{2}x 10^3) for lactone **(-)-14.** U(eq) is defined as one third of the trace of the orthogonalized U^{ij} tensor.

	x	y	z	U(eq)
O(1)	7511(11)	-234(4)	-33(8)	46(2)
O(2)	5125(11)	-176(3)	3219(7)	37(2)
O(3)	3118(10)	696(4)	3481(8)	43(2)
C(1)	7767(18)	275(5)	632(11)	39(3)
C(2)	8120(20)	293(5)	2189(11)	42(3)
C(3)	7423(19)	-330(5)	2902(13)	48(3)
C(4)	4857(16)	484(5)	3237(9)	31(2)
C(5)	7037(14)	827(5)	3063(10)	32(2)
C(6)	8011(17)	911(5)	4473(10)	36(2)
C(7)	6920(20)	1470(5)	5294(13)	46(3)
C(8)	7160(20)	2114(5)	4477(11)	43(3)
C(9)	6280(19)	2109(6)	3020(13)	47(3)
C(10)	6991(16)	1491(5)	2237(9)	32(2)
C(11)	6060(20)	1405(5)	761(13)	47(3)
C(12)	7381(18)	923(5)	-76(10)	41(3)
C(13)	3690(20)	2157(5)	3078(13)	46(3)
C(14)	7090(20)	2726(6)	2287(11)	47(3)

Table S11. Bond lengths [Å] and angles [°] for lactone **(-)-14.**

O(1)-C(1)	1.228(12)
O(2)-C(4)	1.355(12)
O(2)-C(3)	1.505(14)
O(3)-C(4)	1.195(12)
C(1)-C(12)	1.504(14)
C(1)-C(2)	1.508(15)
C(2)-C(3)	1.507(16)
C(2)-C(5)	1.531(14)
C(4)-C(5)	1.543(14)
C(5)-C(6)	1.492(14)
C(5)-C(10)	1.568(14)
C(6)-C(7)	1.546(16)
C(7)-C(8)	1.536(15)
C(8)-C(9)	1.501(15)
C(9)-C(14)	1.528(16)
C(9)-C(10)	1.533(15)
C(9)-C(13)	1.625(17)
C(10)-C(11)	1.538(15)
C(11)-C(12)	1.514(16)
C(4)-O(2)-C(3)	109.2(7)
O(1)-C(1)-C(12)	119.1(9)
O(1)-C(1)-C(2)	123.5(9)
C(12)-C(1)-C(2)	116.6(9)
C(3)-C(2)-C(1)	112.7(9)
C(3)-C(2)-C(5)	102.9(9)
C(1)-C(2)-C(5)	119.6(9)

O(2)-C(3)-C(2)	101.0(8)
O(3)-C(4)-O(2)	118.3(9)
O(3)-C(4)-C(5)	131.6(9)
O(2)-C(4)-C(5)	109.8(8)
C(6)-C(5)-C(2)	113.3(8)
C(6)-C(5)-C(4)	108.4(8)
C(2)-C(5)-C(4)	97.4(8)
C(6)-C(5)-C(10)	111.4(8)
C(2)-C(5)-C(10)	110.2(8)
C(4)-C(5)-C(10)	115.5(7)
C(5)-C(6)-C(7)	111.3(8)
C(8)-C(7)-C(6)	109.0(10)
C(9)-C(8)-C(7)	115.5(9)
C(8)-C(9)-C(14)	107.3(9)
C(8)-C(9)-C(10)	110.7(9)
C(14)-C(9)-C(10)	110.8(9)
C(8)-C(9)-C(13)	109.5(10)
C(14)-C(9)-C(13)	107.5(9)
C(10)-C(9)-C(13)	110.9(8)
C(9)-C(10)-C(11)	115.7(8)
C(9)-C(10)-C(5)	117.9(8)
C(11)-C(10)-C(5)	111.9(8)
C(12)-C(11)-C(10)	110.8(9)
C(1)-C(12)-C(11)	114.7(9)

Symmetry transformations used to generate equivalent atoms:

Table S12. Anisotropic displacement parameters ($Å^2$x 10^3) for **(-)-14.** The anisotropic displacement factor exponent takes the form: -2π^2[h^2 $a^{*2}U^{11}$ + ... + 2 h k a* b* U^{12}]

	U^1	U^{22}	U^{33}	U^{23}	U^{13}	U^{12}
O(1)	40(4)	47(5)	50(5)	-16(4)	-17(4)	7(3)
O(2)	36(4)	29(4)	46(4)	5(3)	12(3)	-7(3)
O(3)	20(4)	43(5)	67(5)	-8(4)	4(3)	6(3)
C(1)	47(6)	34(6)	36(6)	2(4)	0(5)	10(4)
C(2)	59(7)	37(6)	29(5)	-6(4)	0(5)	1(5)
C(3)	60(8)	31(6)	52(7)	2(5)	-11(6)	13(5)
C(4)	39(6)	33(5)	22(4)	-1(4)	-11(4)	-6(4)
C(5)	23(5)	38(6)	36(5)	-2(4)	1(4)	10(4)
C(6)	38(5)	36(6)	33(5)	6(4)	4(4)	1(4)
C(7)	50(6)	40(7)	49(7)	-7(5)	-3(5)	4(5)
C(8)	67(8)	29(6)	33(5)	-7(4)	-11(5)	-2(5)
C(9)	52(7)	38(6)	49(6)	4(5)	-7(5)	-7(5)
C(10)	36(5)	35(6)	24(4)	3(4)	12(4)	-1(4)
C(11)	61(7)	25(5)	54(7)	4(5)	-7(6)	-3(5)
C(12)	58(7)	40(6)	25(5)	-1(4)	-8(5)	9(5)
C(13)	59(7)	30(6)	49(6)	-2(4)	0(5)	14(5)
C(14)	58(7)	53(7)	30(5)	0(5)	4(5)	-9(5)
